# Characterization of Carbapenem-Resistant Enterobacteriaceae Cultured From Retail Meat Products, Patients, and Porcine Excrement in China

**DOI:** 10.3389/fmicb.2021.743468

**Published:** 2021-12-23

**Authors:** Jie Feng, Qian Xiang, Jiangang Ma, Pei Zhang, Kun Li, Ke Wu, Mengru Su, Ruichao Li, Daniel Hurley, Li Bai, Juan Wang, Zengqi Yang

**Affiliations:** ^1^College of Veterinary Medicine, Northwest A&F University, Xianyang, China; ^2^Department of Healthcare Associated Infection Control, Sichuan Provincial People’s Hospital, University of Electronic Science and Technology of China, Chengdu, China; ^3^National Health Commission Key Laboratory of Food Safety Risk Assessment, Food Safety Research Unit (2019RU014) of Chinese Academy of Medical Science, China National Center for Food Safety Risk Assessment, Beijing, China; ^4^Jiangsu Co-innovation Center for Prevention and Control of Important Animal Infectious Diseases and Zoonoses, College of Veterinary Medicine, Yangzhou University, Yangzhou, China; ^5^UCD-Centre for Food Safety, UCD School of Agriculture and Food Science, University College Dublin, Dublin, Ireland

**Keywords:** CRE, *E. coli*, *K. pneumoniae*, *bla*_NDM_, plasmids

## Abstract

The emergence and dissemination of carbapenem-resistant Enterobacteriaceae (CRE) is a growing concern to animal and public health. However, little is known about the spread of CRE in food and livestock and its potential transmission to humans. To identify CRE strains from different origins and sources, 53 isolates were cultured from 760 samples including retail meat products, patients, and porcine excrement. Antimicrobial susceptibility testing was carried out, followed by phylogenetic typing, whole-genome sequencing, broth mating assays, and plasmids analyses. Forty-three *Escherichia coli*, nine *Klebsiella pneumoniae*, and one *Enterobacter cloacae* isolates were identified, each exhibiting multidrug-resistant phenotypes. Genetically, the main sequence types (STs) of *E. coli* were ST156 (*n* = 7), ST354 (*n* = 7), and ST48 (*n* = 7), and the dominant ST of *K. pneumoniae* is ST11 (*n* = 5). *bla*_NDM–5_ (*n* = 40) of *E. coli* and *bla*_KPC–2_ (*n* = 5) were the key genes that conferred carbapenem resistance phenotypes in these CRE strains. Additionally, the *mcr-1* gene was identified in 17 *bla*_NDM_-producing isolates. The *bla*_NDM–5_ gene from eight strains could be transferred to the recipients *via* conjugation assays. Two *mcr-1* genes in the *E. coli* isolates could be co-transferred along with the *bla*_NDM–5_ genes. IncF and IncX3 plasmids have been found to be predominantly associated with *bla*_NDM_ gene in these strains. Strains isolated in our study from different sources and regions tend to be concordant and overlap. CRE strains from retail meat products are a reservoir for transition of CRE strains between animals and humans. These data also provide evidence of the dissemination of CRE strains and carbapenem-resistant genes between animal and human sources.

## Introduction

The abuse of antibiotics has led to a crisis of antibiotic resistance, which has become a formidable threat to public health in China and around the globe. Increasing antibiotic consumption has been accompanied by the emergence of antibiotic-resistant Gram-negative pathogens (Xu et al., 2015). Multidrug-resistant bacteria (MDRs), especially Gram-negative bacteria resistant to carbapenems, known as carbapenem-resistant Enterobacteriaceae (CRE), are the most critical (Wang et al., 2015).

Carbapenems are the favored last-resort antibiotics for treatment of MDR infection. Meropenem (MEM) and imipenem (IMI) are the two most clinically used carbapenems. However, these are increasingly ineffective with the emergence of carbapenemase in CRE. Thus, CRE cause hard-to-treat infections among hospitalized patients (Zhang R. et al., 2017). However, there are relatively few reports on the characteristics of CRE strains from different sources in different provinces in China.

It is acknowledged that the horizontal transfer of plasmids carrying resistance genes is an important contributory factor in the epidemiology of this bacterial ecosystem. Plasmids evolve as an integral part of the bacterial genome, consisting of several extra-chromosomal traits. One of which is resistance genes, which can be exchanged among bacteria of different genera and origins by conjugation. Those expressing a carbapenem-resistant phenotype frequently carry genes encoding resistance to other commonly used antimicrobial drug classes. The use of antimicrobial compounds in human and veterinary medicine also constitutes a risk factor for the selection and dissemination of resistant clones, as well as plasmids containing these resistance genes. To address this problem, it is important to monitor the dissemination of carbapenemase-producing bacteria and characterize these microbes when they arise. Moreover, studies describing the genetic basis of plasmids derived from bacteria recovered from animals and humans are essential.

The aims of this study were (i) to reveal the emergence of CRE isolates cultured from retail meat products, pig feces, and patients in China; (ii) to identify the genes relating to carbapenem resistance; (iii) to characterize the donor isolates by phylogenetic grouping and multi-locus sequence typing (MLST); and (iv) to determine the co-transferred resistance markers by conjugation assays along with S1-nuclease-based plasmid profiles. The epidemiological data presented extend our understanding of this resistance type in humans and animals.

## Materials and Methods

### Strain Isolation and Identification

Between 2012 and 2016, 760 non-duplicate swabs were collected randomly from retail meat products (*n* = 520), porcine excrement (*n* = 68), and clinical samples (stools from patients with intestinal/respiratory diseases)(*n* = 172) from the provinces of Hebei (*n* = 473), Henan (*n* = 201), and Sichuan (*n* = 86) in China. Subsequently, the carbapenem-resistant strains were selected by MacConkey plates containing 2 μg/ml MEM and identified by 16S rRNA gene amplification and sequencing.

### Antimicrobial Susceptibility Testing

According to CLSI and EUCAST^1^ recommendations, the minimal inhibitory concentrations (MICs) of the identified strains were determined for 17 antimicrobial compounds (Supplementary Table 1) conducted using the broth microdilution method. *Escherichia coli* ATCC™ 25922 and *Klebsiella pneumoniae* ATCC™ 700603 were included as quality-control strains. Susceptibility/resistance was interpreted according to the CLSI standard 2020 (Clinical and Laboratory Standards Institute [CLSI], 2020). MDR organisms were defined as acquired non-susceptibility to at least one agent in three or more antimicrobial categories. Extensively drug-resistant (XDR) organisms were defined as those that acquired non-susceptibility to all but two drug classes. Pan-drug-resistant (PDR) organisms were defined as those that acquired non-susceptibility to all drug classes.

### Pulsed-Field Gel Electrophoresis

All CRE strains were classified by *Apa*I nuclease-digested pulsed-field gel electrophoresis (PFGE; Ribot et al., 2006). Pulsotypes (PTs) were compared using BioNumerics version 6.6 (Applied Math, Sint-Martens-Latem, Belgium). PTs were interpreted using an 85% similarity cut-off. The *Xba*I nuclease-digested *Salmonella enterica* serotype Braenderup H9812 was used as a size marker.

### Whole-Genome Sequencing, Phylogenetic Analysis, and Annotation

Genomic DNA was sequenced using the Illumina HiSeq 2500 platform (Illumina, San Diego, CA, United States). Open reading frames (ORFs) were designated using the Rapid Annotation using Subsystem Technology (RAST) annotation pipeline^2^. The ResFinder 4.1^3^ and PlasmidFinder 2.1^4^ databases from the Center for Genomic Epidemiology were also used to identify genomic features of interest. All the *E. coli* and *K. pneumoniae* isolates were subsequently classified for bacterial source tracking analysis by implementing a core genome SNP (cgSNP) strategy that was integrated with the BacWGSTdb 2.0 server^5^. An *E. coli* ST10 strain MG1655 (NCBI reference sequence: U00096) and a *K. pneumoniae* ST11 strain HS11286 (NCBI reference sequence: CP003200) were used as the reference genomes for all *E. coli* and *K. pneumoniae* sequence analyses, respectively. The resolution power of cgSNP analyses was assessed using Simpson’s diversity index. A phylogenetic tree was constructed using the resulting SNPs with recombination regions removed using the maximum parsimony algorithm. Annotation for each isolate and tree embellishment were visualized using iTOL^6^. The Global Optimal eBURST (goeBURST) full MST algorithm was used to compare against the PubMLST database, to highlight the potential relationships of different strain isolates and sequence types (STs) (Lu et al., 2020). The genetic information and evolutionary characteristics of all strains were considered. Nucleotide sequence analyses of 16S rRNA genes of all strains were conducted with MEGA (Lu et al., 2020). Virulence determinants were investigated by aligning the reads to the Virulence Factor Database (VFDB)^7, 8^. In addition, MLST was defined as previously described (Francisco et al., 2009).

### Conjugation Experiments and S1-Pulsed-Field Gel Electrophoresis

After sequencing, it was found that all strains carried plasmids, and carbapenem resistance genes were located on their plasmids. Then, all isolates were selected and analyzed individually for their ability to transfer the IPI/cefotaxime (CTX) resistance phenotype to a rifampicin-resistant, plasmid-free *E. coli* recipient (26R 793 rif^R^), which was performed as previously described (Milton et al., 1996). In brief, donor and recipient cells were mixed at a ratio of 10:1 and spotted on LB at 37°C overnight. Transconjugants were selected on Mueller-Hinton (MH) agar plates containing rifampicin (100 mg/L) and IPI (1 mg/L) or CTX (4 mg/L). Susceptibility tests were performed on the transconjugants to confirm the resistance profile, followed by S1-PFGE to confirm plasmid transfer.

S1-pulsed-field gel electrophoresis analysis of successfully transferred CRE strains and transconjugants was performed as previously described (Bret et al., 1995). Briefly, plugs were made using a mid-log bacterial cell suspension in Tris-EDTA (TE) buffer and further treated with S1 nuclease (Thermo Fisher Scientific, Waltham, MA, United States). Switch times were ramped between 3 and 36 s for 18.5 h at 6 V/cm. The gels were stained with 1 mg/ml ethidium bromide (Sigma-Aldrich, St. Louis, MO, United States) in 0.5× TBE for 30 min in the dark and then washed with distilled water for 90 min to remove excess stains. *Salmonella enterica* serotype Braenderup H9812 was used as a size marker.

### Nucleotide Sequence Accession Numbers

The nucleotide sequences of all 53 CRE strains have been submitted to the NCBI BioProject database^9^ under accession numbers SRR11517132–SRR11517184.

## Results

### Phenotypes of the 53 Carbapenem-Resistant Enterobacteriaceae Strains

In this study, a total of 53 CRE strains were isolated from 760 samples including retail meat products, porcine excrement, and patients. HK and SK in strain names mean they were identified from samples collected from Zhengzhou in Henan province and Mianyang in Szechwan province, respectively. And SJ and SJE strains were collected from Shijiazhuang in Hebei province. Thirty-six isolates were cultured from retail meat products from Shijiazhuang and Zhengzhou, including 30 *E. coli*, 5 *K. pneumoniae*, and 1 *Enterobacter cloacae*. Six *E. coli* isolates were from the porcine excrement only from Mianyang. Twelve isolates were from respiratory secretions of patients with respiratory infection, including seven *E. coli* and four *K. pneumoniae* strains (Supplementary Table 2) from Shijiazhuang and Zhengzhou.

Susceptibility testing confirmed that 53 CRE strains showed resistance to both IPI and MEM. Few isolates showed resistance to polymyxin B (*n* = 3, 5.7%) and amikacin (*n* = 14, 26.4%), while the majority was resistant to ampicillin (*n* = 53, 100%), cefepime (*n* = 53, 100%), cefotaxime (*n* = 53, 100%), ceftazidime (*n* = 52, 98.1%), tetracycline (*n* = 50, 94.3%), and nalidixic (*n* = 50, 94.3%) (Table 1). Of the 53 strains, 46 (86.79%) were XDR Enterobacteriaceae (resistant to all we tested but polymyxins). And the other seven strains were PDR Enterobacteriaceae.

**TABLE 1:** Minimal inhibitory concentration results of 17 antibiotics of all the isolates in this study.

Strains	MIC (mg/L)																
	AMP	CTX	FEP	CAZ	IMI	MEM	GEN	AMI	STR	SUL	SXT	CHL	AZI	TET	NAL	CIP	PB
HK1	>64	>8	>16	>16	>8	>4	32	≤4	>32	>512	>8/152	≤2	4	32	>64	0.125	≤0.5
HK2	>64	>8	>16	>16	>8	>4	>32	>128	>32	>512	>8	>64	16	>32	>64	8	2
HK4	>64	>8	>16	>16	8	>4	>32	>128	>32	>512	>8/152	>64	16	>32	>64	16	≤0.5
HK5	>64	>8	>16	>16	8	>4	32	≤4	>32	256	≤0.25	>64	≤2	32	>64	32	≤0.5
HK9	>64	>8	>16	>16	>8	>4	≤1	≤4	>32	128	>8	>64	8	>32	>64	0.25	≤0.5
HK11	>64	>8	>16	>16	>8	>4	>32	≤4	>32	256	>8	>64	64	>32	>64	16	≤0.5
HK13	>64	>8	>16	>16	4	>4	16	≤4	>32	64	>8	>64	≤2	>32	>64	>32	4
HK15	>64	>8	>16	>16	4	>4	>32	>128	>32	512	>8	>64	32	>32	>64	>32	≤0.5
HK16	>64	>8	>16	>16	8	>4	16	≤4	>32	256	>8	>64	≤2	>32	>64	>32	2
HK17	>64	>8	>16	>16	8	>4	32	≤4	>32	512	>8	>64	≤2	>32	16	4	≤0.5
HK18	>64	>8	>16	>16	4	>4	≤1	≤4	≤4	≤32	0.5	32	4	4	>64	>32	4
HK20	>64	>8	>16	>16	8	>4	16	≤4	>32	>512	>8	>64	16	>32	>64	>32	2
SJ1	>64	>8	>16	>16	>8	>4	16	≤4	8	128	>8	16	>64	>32	>64	>32	≤0.5
SJ2	>64	>8	>16	>16	>8	>4	>32	≤4	>32	512	>8	>64	16	>32	>64	32	≤0.5
SJ3	>64	>8	>16	>16	>8	>4	32	≤4	16	512	>8	>64	8	>32	>64	32	≤0.5
SJ4	>64	>8	>16	>16	>8	>4	>32	≤4	32	>512	>8	>64	8	>32	>64	32	≤0.5
SJ6	>64	>8	>16	>16	>8	>4	>32	8	>32	512	>8	>64	4	>32	>64	>32	≤0.5
SJ7	>64	>8	>16	>16	>8	>4	>32	≤4	16	256	>8	>64	8	>32	>64	32	≤0.5
SJ10	>64	>8	>16	>16	8	>4	>32	≤4	>32	256	>8	>64	32	>32	>64	>32	2
SJ12	>64	>8	>16	>16	8	>4	32	≤4	≤4	>512	>8/152	>64	8	>32	>64	32	≤0.5
SJ15	>64	>8	>16	>16	2	4	32	≤4	16	>512	>8/152	>64	8	>32	>64	32	≤0.5
SJ16	>64	>8	>16	>16	>8	>4	>32	>128	>32	>512	>8/152	>64	16	>32	>64	32	≤0.5
SJ19	>64	>8	>16	>16	4	>4	16	≤4	16	>512	>8/152	>64	32	>32	>64	16	2
SJ20	>64	>8	>16	>16	>8	>4	>32	≤4	16	>512	>8/152	>64	8	>32	>64	16	≤0.5
SJ21	>64	>8	>16	>16	>8	>4	16	8	16	>512	>8/152	>64	32	>32	>64	>32	2
SJ22	>64	>8	16	>16	4	>4	32	≤4	>32	>512	>8/152	8	32	>32	≤4	0.25	1
SJ23	>64	>8	>16	>16	>8	>4	>32	16	>32	>512	>8/152	>64	4	>32	>64	>32	2
SJ25	>64	>8	>16	>16	>8	>4	>32	>128	16	>512	>8/152	16	>64	>32	>64	>32	≤0.5
SJ26	>64	>8	>16	>16	>8	>4	>32	>128	16	>512	≤0.25/4.75	4	64	>32	>64	32	≤0.5
SJ27	>64	>8	>16	>16	>8	>4	>32	>128	32	>512	>8/152	4	64	>32	>64	>32	≤0.5
SJ28	>64	>8	>16	>16	>8	>4	>32	>128	16	>512	>8/152	8	64	>32	>64	>32	≤0.5
SJ29	>64	>8	>16	>16	>8	>4	32	16	>32	>512	>8/152	>64	>64	>32	>64	>32	2
SJ30	>64	>8	>16	>16	>8	>4	>32	>128	>32	>512	>8/152	>64	8	32	>64	>32	1
SJ31	>64	>8	>16	2	8	>4	>32	>128	>32	>512	≤0.25/4.75	≤2	>64	>32	>64	4	>16
SJ34	>64	>8	>16	>16	>8	>4	>32	32	>32	>512	>8/152	>64	16	>32	>64	>32	4
SJ36	>64	>8	>16	>16	>8	>4	>32	>128	16	>512	>8/152	4	64	>32	>64	>32	≤0.5
SJE39	>64	>8	>16	>16	2	4	≤1	≤4	16	>512	>8/152	>64	≤2	>32	>64	16	4
SK1	>64	>8	>16	>16	>8	>4	>32	≤4	>32	256	>8	>64	≤2	>32	>64	8	≤0.5
SK2	>64	>8	16	>16	4	>4	32	≤4	>32	>512	>8/152	>64	≤2	32	>64	8	1
SK3	>64	>8	>16	>16	8	>4	>32	<4	>32	>512	>8	>64	<2	>32	>64	8	<0.5
SK4	>64	>8	>16	>16	>8	>4	>32	<4	16	512	>8	>64	4	>32	>64	32	<0.5
SK5	>64	>8	16	>16	8	>4	32	<4	>32	256	>8	>64	4	>32	>64	8	<0.5
SK7	>64	>8	16	>16	4	>4	≤1	≤4	≤4	≤32	≤0.25/4.75	4	≤2	32	≤4	≤0.03	≤0.5
HK14	>64	>8	>16	>16	>8	>4	<1	16	>32	>512	>8	>64	4	>32	>64	>32	16
HK19	>64	>8	>16	>16	>8	>4	<1	<4	<4	<32	>8	>64	64	>32	>64	>32	<0.5
SJ9	>64	>8	>16	>16	>8	>4	32	<4	>32	>512	>8	>64	8	>32	32	8	<0.5
SJ11	>64	>8	>16	>16	>8	>4	>32	>128	32	<32	<0.25	32	32	2	>64	>32	<0.5
SJ13	>64	>8	>16	>16	8	>4	32	≤4	>32	>512	>8/152	>64	64	>32	32	4	≤0.5
SJ17	>64	>8	>16	>16	8	>4	≤1	≤4	≤4	>512	>8/152	>64	64	>32	>64	32	≤0.5
SJ18	>64	>8	>16	>16	>8	>4	>32	>128	8	≤32	≤0.25/4.75	32	16	2	>64	32	≤0.5
SJ24	>64	>8	>16	>16	>8	>4	32	≤4	≤4	>512	>8/152	>64	32	>32	>64	32	≤0.5
SJ32	>64	>8	>16	>16	4	>4	>32	>128	>32	>512	>8/152	>64	32	>32	>64	>32	1
SJE44	>64	>8	>16	>16	8	>4	4	≤4	≤4	>512	≤0.25/4.75	>64	>64	>32	>64	>32	16

*Antibiotic names and corresponding acronyms: AMP, ampicillin; CTX, cefotaxime; FEP, cefepime; CAZ, ceftazidime; IMI, imipenem; MEM, meropenem; GEN, gentamicin; AMI, amikacin; STR, streptomycin; SUL, sulfonamides; SXT, trimethoprim–sulfamethoxazole; CHL, chloramphenicol; AZI, azithromycin; TET, tetracycline; NAL, nalidixic acid; CIP, ciprofloxacin; PB, polymyxin B.*

### Genotypes of the *Escherichia coli* and the *Klebsiella pneumoniae* Strains

**FIGURE 1:**
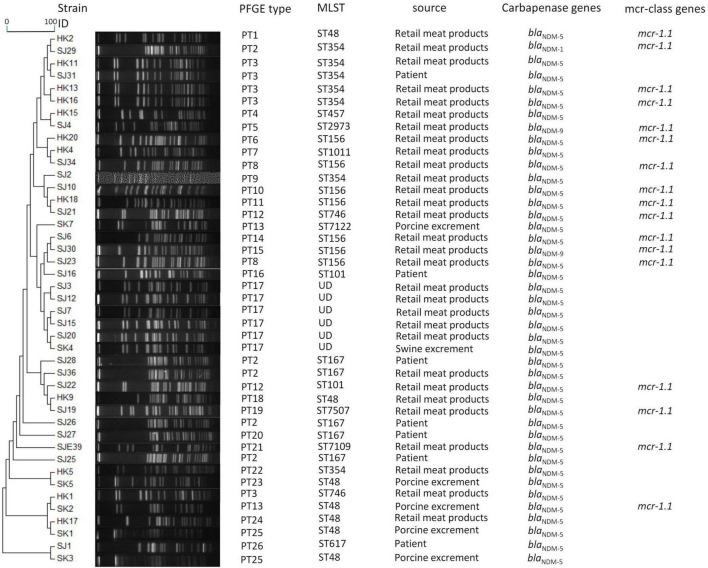
Dendrogram of PTs of chromosomal DNA restriction fragments from 43 *E. coli* isolates in the present study. Allelic profiles, PTs, STs, source information, carbapenemase genes, and *mcr*-class genes. The *bla*_NDM–5_+*mcr-1* genotype was identified in ST48, ST101, ST156, ST354, ST7109, and ST7507. The *bla*_NDM–5_+*bla*_OXA–1_+*mcr-1* genotype was identified in ST746. The *bla*_NDM–1_+*mcr-1* genotype was identified in ST354. *bla*_NDM–9_+*mcr-1* was identified in ST156 and ST2973.

There are 14 different STs in the 43 *E. coli* isolates, with 30 retail meat product isolates clustered in 13 STs (ST10, ST48, ST101, ST156, SST167, ST354, ST457, ST746, ST1011, T2973, ST7109, ST7122, and ST7507), 7 *E. coli* isolates from patients clustered in 4 STs (ST101, ST167, ST354, and ST617), and 6 isolates from porcine excrement clustered in 1 ST (ST48) (Figure 1). Of 43 *E. coli* strains, 60.47% (Thanassi et al., 2012) was identified as belonging to ST156 (7/43), ST354 (7/43), ST48 (7/43), and ST167 (5/43). ST156 strains were mainly isolated from samples of retail meat products, while ST167 strains were isolated principally from samples of patients. Five of seven *E. coli* strains’ STs from patients were compatible with *E. coli* from retail meat products, particularly ST167 and ST354. In contrast, ST167 *E. coli* was mainly distributed in patients, and ST354 *E. coli* was mostly distributed in retail meat products. A total of 141,114 SNPs (minimum: 106; maximum: 70,504) were identified among the core genomes of the 43 isolates, on the basis of which the isolates could be allocated into least six major clades (Supplementary Figures 1, 2). The first clade includes 12 *E. coli*. Seven of them from retail meat products and five from patients. Clade 2 comprised six ST167 *E. coli* isolates, of which one was isolated from retail meat products and five isolated from patients. Clade 3 includes eight isolates, of which six isolates were from retail meats. Just one ST1011 *E. coli* isolate from retail meat products was identified as clade 4. Six unclassifiable isolates were included in clade 5.

**FIGURE 2:**
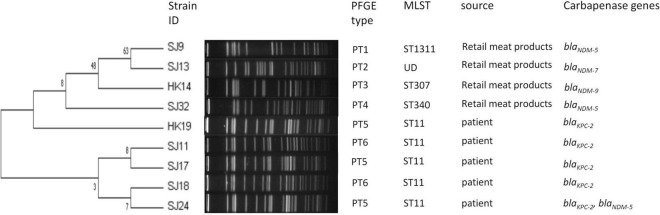
Dendrogram of PTs of chromosomal DNA restriction fragments from nine *K. pneumoniae* isolates in the present study. Allelic profiles, PTs, STs, source information, and carbapenemase genes.

Nine *K. pneumoniae* isolates covered five STs. The most prevalent ST was ST11 (5/9, 55.6%), which was all from patients (Figure 2). The distribution of STs in retail meat products showed diversity including ST307, ST340, and ST1311, and the ST of SJ13 was not defined. A total of 49,975 SNPs (minimum: 51; maximum: 24,889) were identified among the core genomes of the nine isolates, on the basis of which the isolates could be allocated into least three major lineages (Supplementary Figures 3, 4). Clades 1 and 2 comprised isolates from retail meat products, and clade 3 from patients.

**FIGURE 3:**
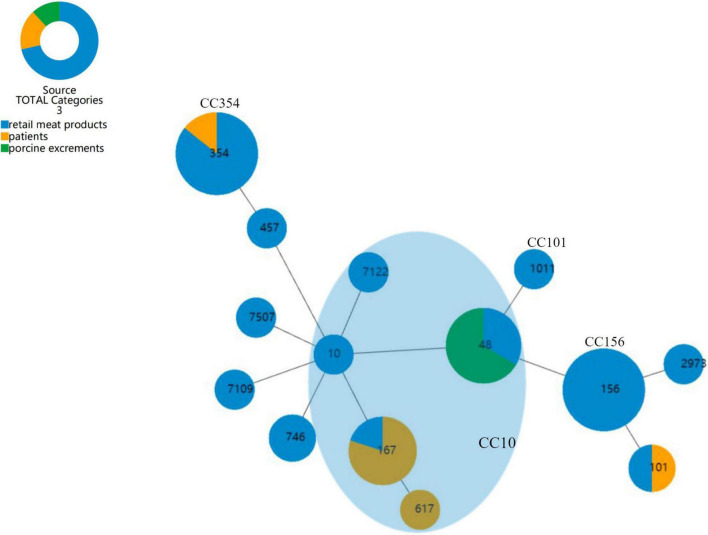
goeBURST full MST analysis of 14 STs of *E. coli*. Each circle represents an ST, and the size of the circle reflects the frequency of each ST in the dataset; the number of different alleles is presented as STs, and the line between each circle is presented as distance labels (label edges with level value). CC represents a unique clonal complex. The shaded area represents the clonal complex 10.

We aimed to discover if there was a trend in the dissemination of CR-producing *E. coli*, so that the risks of transmission of different strains could be estimated. The goeBURST full MST algorithm used in the present study of *E. coli* categorized all STs into four clone complexes (CC), CC10, CC101, CC156, and CC354 (Figure 3). CC10 was the biggest group which harbored 5 STs and 15 *E. coli* strains. Next is CC354, which harbored ST354 with only seven *E. coli* strains, and CC156, which included six ST156 *E. coli* strains, all of which were collected from retail meat products. SJ16 of patients and SJ22 of retail meat products are ST101 *E. coli* belonging to CC101.

As for PTs, there were 26 PTs observed for 43 *E. coli*, among which 30 were *E. coli* isolates from retail meat products and clustered in 20 PTs, 7 *E. coli* isolates from patients and clustered in 5 PTs, and 6 isolates from porcine excrement and clustered in 5 PTs. A crossover between patients and retail meat products was observed in PT2 and PT3; PT2 *E. coli* (SJ25, SJ26, and SJ28) from patients belonged to ST167, while PT2 *E. coli* from retail meat products belonged to ST167 (SJ36) and ST354 (SJ29); similarly, PT3 *E. coli* from patients and retail meat products respectively belonged to ST354 (SJ31) as well as ST354 (HK11, HK13, and HK16) and ST746 (HK1). To some extent, strains of ST167 and ST354 had a certain similarity and relationship, both of them being associated with PT2. Strains from both porcine excrement (SK4) and retail meat products (SJ3, SJ12, SJ7, SJ15, and SJ20) were associated with PT17, but their STs were not definite (Figure 1). Nine *K. pneumoniae* isolates were divided into six PTs: three PT5 strains from patients (HK19, SJ17, and SJ24) and two PT6 strains from patients (SJ11 and SJ18) belonged to ST11 *K. pneumoniae*; the other four isolates from retail meat products of different STs each had four different PTs (Figure 2).

### Resistance Genes and Virulence Genes of the 53 Carbapenem-Resistant Enterobacteriaceae Strains

*bla*_NDM_ was the most common carbapenemase gene type among the CRE strains, including *bla*_NDM–5_ (42/53, 79.25%), *bla*_NDM–9_ (4/53, 7.55%), *bla*_NDM–1_ (1/53, 1.89%), and *bla*_NDM–7_ (1/53, 1.89%), suggesting *bla*_NDM_ as the key gene mediating the development of the carbapenem resistance phenotype in this study. The *bla*_NDM–5_ gene was found in 39 *E. coli* (26 strains from retail meat products, 6 from porcine excrement, and 7 from patients) and 3 *K. pneumoniae* [2 strains from retail meat products and 1 strain (SJ24) from patients]. Three *bla*_NDM–9_ from *E. coli* and one *bla*_NDM–9_ from *K. pneumoniae* were all from retail meat products, as well as *bla*_NDM–1_ from *E. coli* and *bla*_NDM–7_ from *K. pneumoniae*.

The gene encoding *K. pneumoniae* carbapenemase (KPC) has been detected in five ST11 *K. pneumoniae* isolates collected in the hospital, with each being *bla*_KPC–2_. Since its identification in North Carolina, United States, in 1996, in a clinical *K. pneumoniae* isolate, the epidemiology of *bla*_KPC_ has expanded globally, especially the clonal group (CG) 258, which includes the lineages ST258 and ST11. SJ24, carrying *bla*_NDM–5_ and *bla*_KPC–2_ simultaneously, had nine different plasmid replicon types, which was the largest number of plasmid replicon types in this study.

All isolates harbored beta-lactamase gene(s), aminoglycosides gene(s), and sulfonamide resistance gene(s). Most isolates harbored fluoroquinolone resistance genes(s), fosfomycin resistance gene(s), macrolide resistance gene(s), phenicol resistance gene(s), tetracycline resistance gene(s), and trimethoprim resistance gene(s) (Supplementary Table 3). Additionally, a few isolates harbored streptogramin B, polymyxin B, and/or rifampicin resistance genes. One out of the four *K. pneumoniae* from meat samples harbored the AmpC beta-lactamase *bla*_CMY–2_. Four *E. coli* harbored a macrolide–lincosamide–streptogramin B resistance gene [*erm*(42) or *erm(B)*].

In our study, the polymyxin B resistance gene *mcr-1* was detected in 17/43 (40.5%) *E. coli* isolates, coexisting with *bla*_NDM_ (*bla*_NDM–5_ and *mcr-1* 14/17, *bla*_NDM–1_ and *mcr-1* 1/17, and *bla*_NDM–9_ and *mcr-1* 2/17). Only one came from porcine excrement (harboring *bla*_NDM–5_ and *mcr-1*); the rest of the 17 strains were all collected from retail meat products (Figure 1).

Virulence genes have also been tallied and collated (Supplementary Table 4). Forty-six, 27, and 15 virulence genes were detected from the *E. coli* strains of retail meat products, patients, and porcine excrement, respectively. Among the 16 virulence genes which represent the main categories of virulence determinants (*sfa*, *pap*C, *sep*A, *etr*A, *aer*, *fea*G, *fsa*A, *eae*A, *rfc*, *cnf*1, *hly*A, *elt*A, *est*A, *exh*A, *stx*1, and *stx*2), we only detected the *pap*C gene in 10 *E. coli*, which encode the protein of pilus associated with pyelonephritis and belong to ExPEC. In addition, *irp2* (iron-repressible protein gene) was detected from 6 *E. coli*, *cva* (colicin V plasmid operon gene) was detected from 17 *E. coli*, *ast*A (enteroaggregative toxin gene) was detected from 12 *E. coli*, *tsh* (temperature-sensitive hemagglutinin gene) was detected from 1 *E. coli*, *iuc*D (ferric aerobactin receptor gene) was detected from 22 *E. coli*, and *iss* (increased serum survival protein gene) was detected from 27 *E. coli*.

Virulence factor profiling of the nine *K. pneumoniae* isolates revealed the presence of 98 virulence factors. The *fim* gene cluster (*fim*ABC, etc.), *mrk* genes (A, B, C, D, F, H, I, and J), *fep* genes (A, B, C, D, and G), *ent* genes (A, B, C, D, E, F, and S), *tss* genes (A, B, C, D, F, G, H, I, J, K, L, and M), *iro* genes (D, E, and N), *ybt* genes (A, E, P, O, S, T, U, and X), *iuc* genes (A and C), *iut*A gene, and *rmp*A gene were highly represented.

### Horizontal Transfer of Antimicrobial Resistance and Associated Determinants

None of the nine *K. pneumoniae* plasmids and one *E. cloacae* plasmid could be transferred by conjugation in three attempts.

**FIGURE 4:**
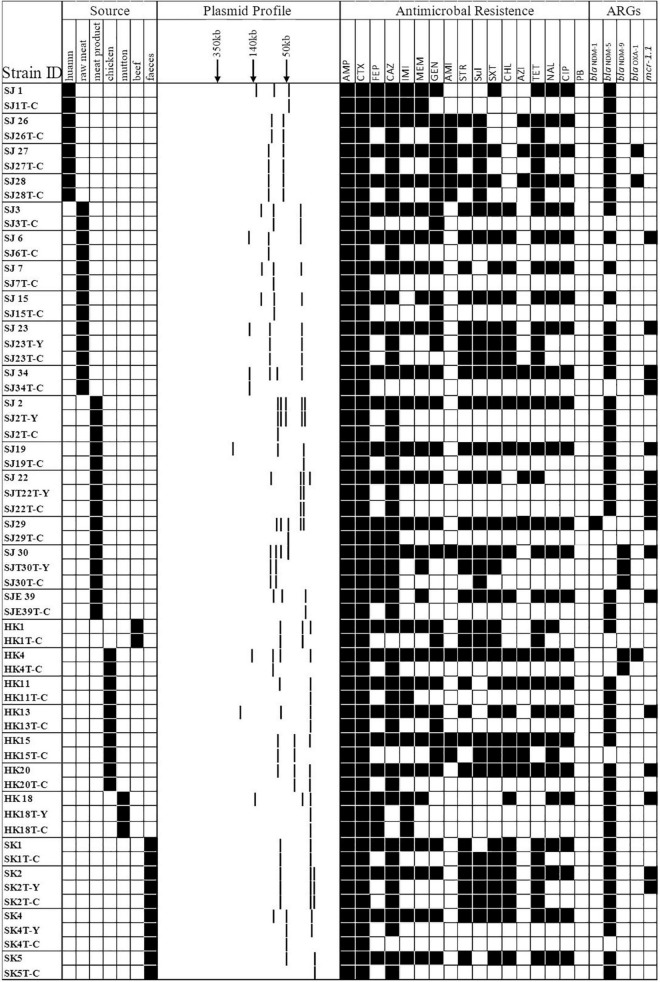
A heat map showing the comparison of donors and the resultant transconjugants, characterized on the basis of the summary of sources; S1-nuclease plasmid profiles of transconjugants; antimicrobial resistance profile; carbapenem-resistant gene(s) and *mcr-1* gene. Y and C in the first column typify the transconjugant from the IPI group and CTX group, respectively. Black squares shown indicate a feature present in that isolate, denoting its original source, its corresponding antimicrobial resistance profile, and the carbapenem-resistant and *mcr-1* gene(s) profile. White squares denote features that are lacking in the corresponding bacterial isolate.

Forty-three *E. coli* strains (seven strains from patients, six from porcine excrement, and the rest from retail meat products) were tested for their ability to transfer IPI and CTX resistance phenotypes by conjugation. A total of 34 transconjugants were obtained. All the transconjugants were resistant to AMP and CTX. Of 34 transconjugants, 30 were resistant to IMI and MEM. After PCR identification, there were 30 transconjugants harboring the *bla*_NDM_ gene; there were 8 transferred donor strains without *bla*_CTX–M_ genes but whose transconjugants were resistant to CTX, and their resistance to CTX may be mediated by other ESBLs. *mcr*-1 was detected in four transconjugants, of which two were recovered from the same donor (SJ22 carries *mcr-1*, *bla*_NDM–5_, and *bla*_TEM–1B_) (Figure 4 and Table 2).

**TABLE 2:** Characteristics of transconjugants.

Strains	Genes detected by PCR		MIC (mg/L)																
	*bla*_NDM_	*mcr-1*	AMP	CTX	FEP	CAZ	IMI	MEM	GEN	AMI	STR	Sul	SXT	CHL	AZI	TET	NAL	CIP	PB
SJ7T-C			>64	4	2	≤0.5	≤0.25	≤0.06	8	≤4	≤4	≤32	≤0.25/4.75	4	≤2	≤1	≤4	≤0.03	≤0.5
SJ2T-C	+		>64	>8	4	>16	>8	>4	≤1	≤4	≤4	≤32	≤0.25/4.75	4	8	≤1	≤4	≤0.03	≤0.5
SJ3T-C			>64	>8	4	1	≤0.25	≤0.06	16	16	≤4	≤32	≤0.25/4.75	4	≤2	≤1	≤4	≤0.03	≤0.5
SJ1T-C	+		>64	>8	>16	>16	8	>4	≤1	≤4	≤4	≤32	≤0.25/4.75	4	≤2	≤1	≤4	≤0.03	≤0.5
SJ10T-C	+		>64	>8	8	>16	4	4	≤1	≤4	8	≤32	≤0.25/4.75	≤2	8	≤1	≤4	≤0.03	≤0.5
SJ15T-C			>64	8	1	≤0.5	≤0.25	≤0.06	16	≤4	≤4	≤32	≤0.25/4.75	4	≤2	≤1	≤4	≤0.03	≤0.5
SJ16T-C	+		>64	>8	1	2	0.5	≤0.06	8	≤4	>32	>512	≤0.25/4.75	>64	≤2	>32	≤4	≤0.03	≤0.5
SJ19T-C	+		>64	>8	4	>16	4	4	≤1	≤4	≤4	≤32	≤0.25/4.75	4	≤2	≤1	≤4	≤0.03	≤0.5
SJ22T-C	+	+	>64	>8	4	>16	4	4	≤1	≤4	≤4	≤32	≤0.25/4.75	4	≤2	≤1	≤4	≤0.03	4
SJ23T-C	+		>64	>8	4	>16	>8	>4	8	≤4	>32	>512	>8/152	>64	≤2	>32	≤4	≤0.03	≤0.5
SJ26T-C	+		>64	>8	4	>16	>8	>4	>32	>128	≤4	512	≤0.25/4.75	4	8	>32	≤4	≤0.03	≤0.5
SJ27T-C	+		>64	>8	8	>16	>8	>4	>32	>128	≤4	512	≤0.25/4.75	4	4	>32	≤4	≤0.03	≤0.5
SJ28T-C	+		>64	>8	4	>16	>8	>4	32	>128	8	512	>0.25/4.75	4	8	>32	≤4	≤0.03	≤0.5
SJ29T-C			>64	>8	16	16	0.5	≤0.06	≤1	≤4	≤4	≤32	≤0.25/4.75	4	≤2	≤1	≤4	≤0.03	≤0.5
SJ30T-C	+		>64	>8	16	>16	>8	>4	≤1	≤4	8	>512	≤0.25/4.75	4	8	≤1	≤4	≤0.03	≤0.5
SJ31T-C	+		>64	>8	8	>16	4	>4	32	>128	>32	512	2/38	8	16	≤1	≤4	32	≤0.5
SJ32T-C	+		>64	>8	>16	>16	4	>4	>32	≤4	>32	>512	>8/152	16	64	>32	>64	>32	≤0.5
SJ34T-C		+	>64	8	1	2	0.5	≤0.06	≤1	≤4	≤4	≤32	≤0.25/4.75	8	≤2	≤1	≤4	≤0.03	4
SJE39T-C	+		>64	>8	4	>16	4	4	≤1	≤4	≤4	≤32	≤0.25/4.75	4	≤2	≤1	≤4	≤0.03	≤0.5
SK1T-C	+		>64	>8	4	>16	>8	>4	8	≤4	>32	>512	>8/152	>64	≤2	>32	≤4	≤0.03	≤0.5
SK2T-C	+		>64	>8	4	>16	4	>4	8	≤4	>32	>512	>8/152	>64	≤2	32	≤4	≤0.03	≤0.5
SK4T-C			>64	>8	4	1	≤0.25	≤0.06	8	≤4	≤4	≤32	≤0.25/4.75	4	≤2	≤1	≤4	≤0.03	≤0.5
SK5T-C	+		>64	>8	4	>16	4	>4	≤1	≤4	≤4	≤32	≤0.25/4.75	4	≤2	≤1	≤4	≤0.03	≤0.5
HK1T-C			>64	>8	2	4	≤0.25	≤0.06	16	≤4	32	>512	>8/152	4	8	>32	≤4	≤0.03	≤0.5
HK4T-C	+		>64	>8	8	>16	4	>4	≤1	≤4	≤4	≤32	≤0.25/4.75	4	8	≤1	≤4	≤0.03	≤0.5
HK11T-C	+		>64	>8	4	>16	>8	>4	≤1	≤4	≤4	≤32	≤0.25/4.75	4	≤2	≤1	≤4	≤0.03	≤0.5
HK13T-C	+		>64	>8	4	>16	4	4	≤1	≤4	≤4	≤32	≤0.25/4.75	4	≤2	≤1	≤4	≤0.03	≤0.5
HK14T-C	+		>64	>8	8	>16	8	4	16	16	>32	512	>8/152	>64	8	>32	≤4	≤0.03	≤0.5
HK15T-C			>64	>8	4	8	≤0.25	≤0.06	>32	>128	≤4	≤32	≤0.25/4.75	4	8	≤1	≤4	≤0.03	≤0.5
HK18T-C	+		>64	>8	4	>16	4	>4	≤1	≤4	≤4	≤32	≤0.25/4.75	4	≤2	≤1	≤4	≤0.03	≤0.5
HK20T-C	+		>64	>8	4	>16	8	4	≤1	≤4	≤4	≤32	≤0.25/4.75	4	≤2	≤1	≤4	≤0.03	≤0.5
SJ10T-Y	+		>64	>8	4	>16	8	>4	≤1	≤4	8	≤32	≤0.25/4.75	4	8	≤1	≤4	≤0.03	≤0.5
SJ22T-Y	+	+	>64	>8	8	>16	4	4	≤1	≤4	≤4	≤32	≤0.25/4.75	4	≤2	≤1	≤4	≤0.03	4
SJ30T-Y	+		>64	>8	>16	>16	>8	>4	≤1	≤4	16	>512	4/76	≤2	8	≤1	≤4	≤0.03	≤0.5
SJ32T-Y	+		>64	>8	>16	>16	4	8	>32	≤4	>32	>512	>8/152	32	64	>32	>64	32	≤0.5
HK18T-Y	+		>64	>8	8	>16	4	>4	≤1	≤4	≤4	≤32	≤0.25/4.75	4	≤2	≤1	≤4	≤0.03	≤0.5
SK2T-Y	+	+	>64	>8	4	>16	4	>4	4	≤4	>32	>512	>8/152	>64	≤2	32	≤4	≤0.03	4
SK4T-Y	+		>64	>8	4	>16	>8	>4	8	≤4	≤4	≤32	≤0.25/4.75	4	≤2	≤1	≤4	≤0.03	≤0.5
SJ23T-Y	+		>64	>8	8	>16	>8	4	16	≤4	>32	>512	>8/152	>64	≤2	>32	≤4	≤0.03	≤0.5

*Strains from retail meat products, patients, and porcine excrements are respectively shaded with yellow, blue, and green.*

*Antibiotic names and corresponding acronyms: AMP, ampicillin; CTX, cefotaxime; FEP, cefepime; CAZ, ceftazidime; IMI, imipenem; MEM, meropenem; GEN, gentamicin; AMI, amikacin; STR, streptomycin; SUL, sulfonamides; SXT, trimethoprim–sulfamethoxazole; CHL, chloramphenicol; AZI, azithromycin; TET, tetracycline; NAL, nalidixic acid; CIP, ciprofloxacin; PB, polymyxin B.*

S1-nuclease plasmid analysis revealed that all 27 transferred donor strains contained large detectable plasmids: mostly possessing three plasmids (*n* = 15, 55.56%), two plasmids (*n* = 6, 22.22%), four plasmids (*n* = 4, 14.81%), and five plasmids (*n* = 2, 7.21%). Compared with those found in the S1-nuclease plasmid analysis in donors, the numbers of plasmids carried by 29/34 transconjugants were reduced (Figure 4).

### Plasmid Replicon Types of the *Escherichia coli* and the *Klebsiella pneumoniae* Strains

**FIGURE 5:**
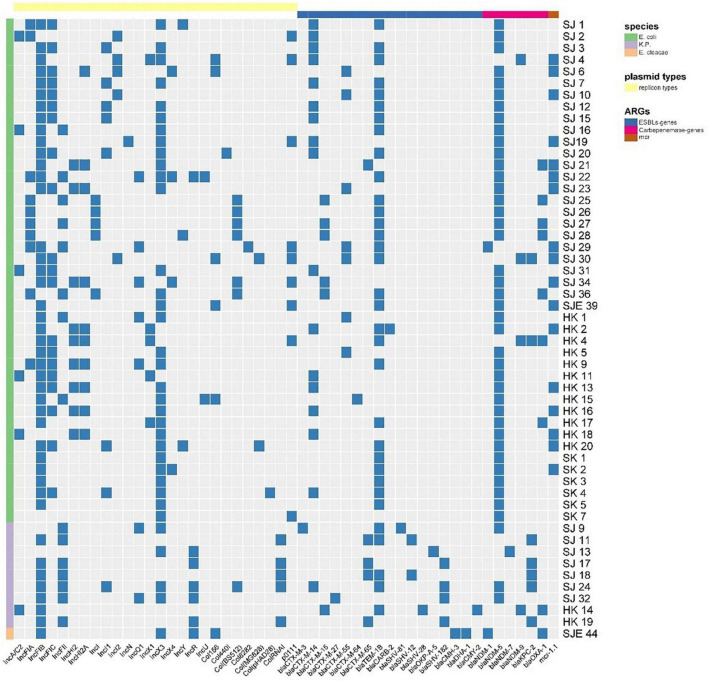
A heat map showing the plasmid replicon types, ESBL genes, CR genes, and *mcr-1* gene of 53 CRE strains. The Col group was split into three groups according to species. In accordance with plasmid replicon types and different categories of ARGs, the raw group was separated into four groups.

In this study, the most prevalent plasmid replicon types in the 43 *E. coli* were IncFIB (35/53, 81.40%, 27 strains were from retail meat product samples, 3 strains were from patient samples, and 5 strains were from porcine excrement), IncX3 (34/43, 79.07%, 25 strains were from retail meat product samples, 3 strains were from patient samples, and 6 strains were from porcine excrement), and IncFIC (19/43, 44.18%, 17 strains were from retail meat product samples, 1 strain was from patient samples, and 1 strain was from porcine excrement). IncFIB (7/9, 5 from patients and 2 from retail meat products), IncFII (as well as IncFIB), IncR (5/9, 3 from patients and 2 from retail meat products), ColRNAI (5/9, all of them collected from patients), and IncX3 (4/9, 1 strain collected from patients and others from retail meat products) were the dominant plasmid replicon types of *K. pneumoniae*. Except for IncFIB and IncX3, the plasmid types of *E. coli* and *K. pneumoniae* were distinct (Figure 5).

### Analysis of Genetic Environments of *bla*_NDM_, *bla*_KPC_, and *mcr-1*

**FIGURE 6:**
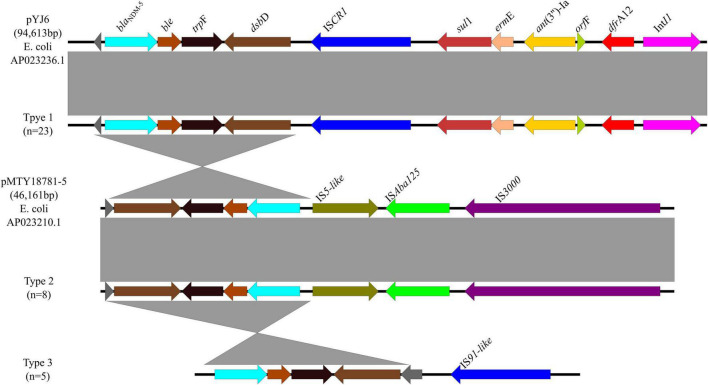
Genetic environments of *bla*_NDM–5_ in plasmids. Arrows indicate the direction of transcription of the genes, and different colors indicate different genes. Regions of ≥99.0% nucleotide sequence identity are shaded gray.

A total of 3 genetic contexts (type 1 to type 3) were found in 36/43 *bla*_NDM–5_-carrying plasmids (Figure 6). Type 1 was found in 14 *bla*_NDM–5_-carrying *E. coli* from retail meats, 5 *bla*_NDM–5_-carrying *E. coli* from porcine excrements, 3 *bla*_NDM–5_-carrying *E. coli* from patients, and 1 *bla*_NDM–7_-carrying *E. coli* from retail meat products. In type 1, IS*CR1* was located downstream of *bla*_NDM–5_, same with plasmid pYJ6 (AP023236.1) of *E. coli*. Type 2 was found in plasmid IncFIB of eight *bla*_NDM–5_-carrying *E. coli* from retail meat products, same with plasmid pMTY18781-5 (AP023210.1) of *E. coli*, which was a 46,161-bp IncFII plasmid. In type 2, IS*Aba125* located between IS*5* and IS*3000* upstream of *bla*_NDM–5_ may play an important role in recombination of plasmids by IS*Aba125* duplication. Type 3 was similar to type 1, except without *sul*1, *ant*(3″)-Ia, and *dfr*A12 genes, which were identified from one *bla*_NDM–5_-carrying *E. coli* from retail meat products and four *bla*_NDM–5_-carrying *E. coli* from patients. The type 1 genomic context *bla*_NDM–5_-*ble*-*trp*F-*dsb*D-IS*CR1*-*sul*1-*erm*E-*ant*(3″)-Ia-*orf*F-*dfr*A12-*IntI1* was the most prevalent *bla*_NDM–5_ genetic environment in this study (63.9%, 23/36).

**FIGURE 7:**
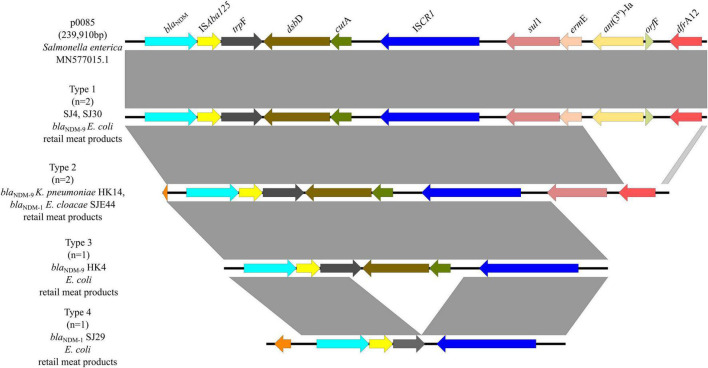
Genetic environments of *bla*_NDM–1/9_ in plasmids. Arrows indicate the direction of transcription of the genes, and different colors indicate different genes. Regions of ≥99.0% nucleotide sequence identity are shaded gray.

In six *bla*_NDM–1/9_-carrying plasmids, there were four genetic contexts (type 1 to type 4) (Figure 7). Type 1 was found in two *bla*_NDM–9_-carrying *E. coli* from retail meat products, same with plasmid p0058 (MN577015.1) of *Salmonella enterica*, which was mainly carried by IncFIB plasmids which obtained IS*Aba125* and IS*CR1* elements. This genomic context was similar to type 1 of *bla*_NDM–5_, except that there was an IS*Aba125* element. In type 2, they carried the same IS elements but without *erm*E, *ant*(3″)-Ia, and *orf*F genes. There were one *bla*_NDM–9_-carrying *K. pneumoniae* and one *bla*_NDM–1_-carrying *E. cloacae* from retail meat products, which belonged to type 2. Types 3 and 4 are similar; neither of them had a *sul*1-*erm*E-*ant*(3″)-Ia-*orf*F-*dfr*A12 genomic context, but type 4 had a truncation *trp*E and did not have *dsb*D and *cut*A genes. One *bla*_NDM–9_-carrying *E. coli* and one *bla*_NDM–1_-carrying *E. coli* from retail meat products belonged to types 3 and 4, respectively.

**FIGURE 8:**
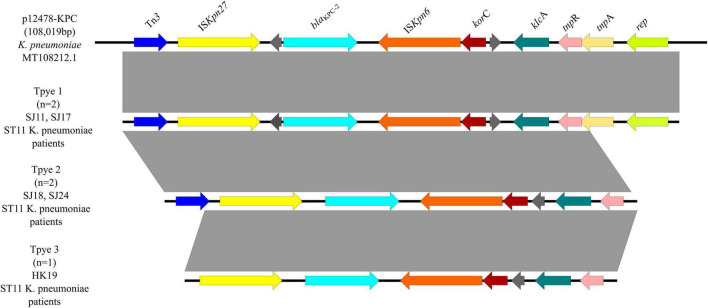
Genetic environments of *bla*_KPC–2_ in plasmids. Arrows indicate the direction of transcription of the genes, and different colors indicate different genes. Regions of ≥99.0% nucleotide sequence identity are shaded gray.

Three genetic contexts (type 1 to type 3) were found in five *bla*_KPC–2_-carrying plasmids (Figure 8), all of which were ST11 *K. pneumoniae* from patients. Two strains were classified as type 1, which was the same with a 108,019-bp plasmid p12478-KPC (MT108212.1). Tn3 and IS*Kpn27* were located upstream of *bla*_KPC–2_, and IS*Kpn6* was located downstream of *bla*_KPC–2_. Type 2, including two ST11 *K. pneumoniae* strains, lacked tnp*A* and *rep* genes compared with type 1. And type 3 lacked a Tn*3* gene compared with type 2, including one ST11 *K. pneumoniae* strain.

**FIGURE 9:**
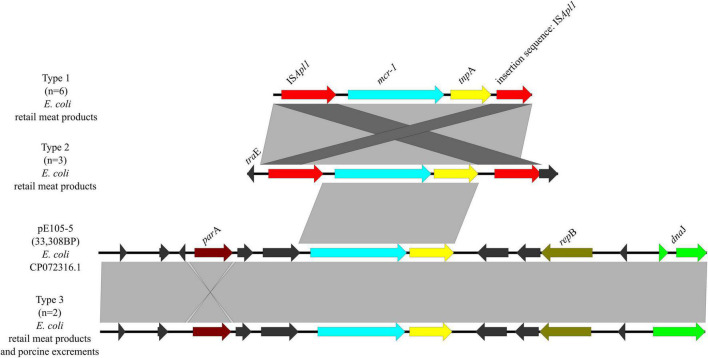
Genetic environments of *mcr-1* in plasmids. Arrows indicate the direction of transcription of the genes, and different colors indicate different genes. Regions of ≥99.0% nucleotide sequence identity are shaded gray.

Three genetic contexts (type 1 to type 3) were found in 11/14 *mcr-1*-carrying plasmids (Figure 9). Type 1 was found in six *mcr-1*-carrying *E. coli* from retail meat products. In type 1, IS*Apl1* was located upstream of *mcr-1* and an insertion sequence of IS*Apl1* in plasmid p0111. In type 2, it was similar to type 1, except that it had *tra*E, which may play an important role in conjugation of plasmids. There were three *mcr-1*-carrying *E. coli* from retail meat products, which belonged to type 2. Type 3 was found in one *mcr-1*-carrying *E. coli* from retail meat products and one *mcr-1*-carrying *E. coli* from porcine excrement, same with plasmid pE105-5 (CP072316.1) of *E. coli*, which was mainly carried by IncX4 plasmids.

## Discussion

Our results demonstrate that isolates from the three origins possess wide genetic variety of carbapenem-resistant gene-producing Enterobacteriaceae. The spread of carbapenem-resistant genes is highly attributed to epidemic and highly transmissible plasmids, which emphasizes the importance of plasmid replicon determination in epidemiological studies. Although the sample size was limited, our study provides an omnibus indicative insight into CRE prevalence in the entire path of animal-derived foods from farm to table. The diversity of AMR genes was high in the isolates from the three source samples positive for CR/ESBL-producing Enterobacteriaceae.

In terms of phenotypes of the 43 *E. coli*, they were identified as XDR or PDR, posing a huge threat to public health. Their multi-resistant patterns ranged from 7 to 17 antibiotics, and most of them were resistant to 13–15 antibiotics. Compared with isolates in the earlier study investigating CR-producing *E. coli* from humans, animals, food, and the environment, the isolates recovered from the three sources in the current study showed broader resistance to multiple antimicrobial groups (Kurittu et al., 2021).

The 43 CR-producing *E. coli* in our study yielded a total of 14 STs and 4 CC, namely, CC10, CC101, CC156, and CC354. CC10 was the largest clonal complex, including 14 strains and 5 STs (ST10, ST48, ST167, ST617, and ST7122), which meant these strains are the main source of infection in the area we studied, carrying a high risk of transmission. Horizontal transmission of plasmids occurs more readily between strains within a clonal complex than between strains from different clonal complexes (Lu et al., 2020). A wide variety of carbapenem-resistant encoding genes were identified, including *bla*_NDM–1_, *bla*_NDM–5_, *bla*_NDM–7_, and *bla*_NDM–9_. Globally, the frequency of *bla*_NDM–1_ is relatively low since its discovery (Wang et al., 2015; Liu et al., 2019), and *bla*_NDM–5_ is often observed in the clinical and edible animal sector monitoring in China (Shen et al., 2018). Moreover, a novel IncX3-type plasmid harboring a *bla*_NDM_ variant (*bla*_NDM–20_) due to three point mutations, compared to *bla*_NDM–1_, was recovered recently in an *E. coli* ST1114 from swine in China, suggesting that food-producing animals could be a reservoir of new carbapenemase genes (Liu et al., 2018).

A study analyzing the persistence and transmission of *bla*_NDM_-producing bacteria from a Chinese poultry farm found that about 20% *bla*_NDM_ carriage also carried either the polymyxin B resistance *mcr-1* or *mcr-8* gene (Taggar et al., 2020). Most *mcr* genes are isolated from animal sources, especially pork, which strongly indicates that pork products are the main source of infection (Lu et al., 2019). Importantly, CRE carrying *mcr-1* may carry additional antibiotic-resistant genes (ARGs) other than CRE without *mcr-1* in normal flora of healthy individuals and animals, especially carbapenem resistance genes (*bla*_NDM_, *bla*_KPC_, etc.) (Li et al., 2018; Shen et al., 2018; Wang et al., 2018). A previous study indicated that acquisition of *mcr-1*- or *bla*_NDM–5_-containing plasmids did not lead to a considerable fitness cost, highlighting the potential for dissemination of *mcr-1* and *bla*_NDM–5_ in Enterobacteriaceae (Zhang Y. et al., 2017). Notably, ST48 *E. coli* is a major member of high-virulence *mcr-1*-positive *E. coli* (MCRPEC) clones, which has increased risk of MCRPEC transmission from livestock to human (Borges et al., 2019).

The finding of resistance genes *bla*_CTX–M–3_, *bla*_CTX–M–14_, *bla*_CTX–M–15_, *bla*_CTX–M–27_, *bla*_CTX–M–55_, *bla*_CTX–M–64_, *bla*_CTX–M–65_, *bla*_TEM–1B_, *bla*_SHV–12_, *bla*_SHV–28_, *bla*_SHV–81_, *bla*_SHV–182_, and *bla*_OXA–1_ being commonly linked to human sources (Livermore et al., 2019) highlights the potential transmission route of AMR *via* food products and emphasizes the importance of hygiene measures. *bla*_OXA–1_ was identified in nine isolates, each also harboring *bla*_NDM_. OXA-1 is a broad-spectrum beta-lactamase belonging to class D oxacillinases, which are epidemiologically important in Enterobacteriaceae (Evans and Amyes, 2014; Codjoe and Donkor, 2017). However, OXA-type carbapenemases (OXA-48 and OXA-23) were not detected in this study. It is interesting to note that nine *bla*_OXA–1_-positive isolates carried other abundant ESBL genes (*bla*_CTX–M–15_, *bla*_CTX–M–65_, *bla*_CMY–2_, *bla*_TEM–1B_, *bla*_SHV–28_, *bla*_CMH–3_, and *bla*_DHA–1_). Notably, four ST167 *E. coli* isolates (one from retail meat products and three from patients) had the same genotype (*bla*_CTX–M–15_, *bla*_NDM–5,_ *bla*_TEM–1B_, and *bla*_OXA–1_), indicating a similar genetic environment conserved in ST167 *E. coli*. Additionally, *bla*_CTX–M–15_ and *bla*_TEM–1B_ were frequently identified along with *bla*_OXA–1_.

Genes conferring resistance to the antimicrobial class macrolide–lincosamide–streptogramin B, as well as macrolides and quinolones, were more frequently observed in the plasmidome samples. The most frequently observed AMR genes related to these three classes were *ermB*, *ermT*, and *ermF* (macrolide–lincosamide–streptogramin B). The higher frequency of these genes in plasmidome samples suggests that they are more frequently found on plasmids in general or on smaller plasmids compared to larger ones (Kirstahler et al., 2021).

There was no significant difference in virulence genes among strains from different regions, but virulence genes carried by strains from different sources were significantly different, even if there is some overlap. It may be that the virulence genes carried by different STs or CCs differed. There are 16 virulence genes which represent the main categories of virulence determinants, including adhesins (*sfa*, *pap*C, *sep*A, *etr*A, *aer*, *fea*G, *fsa*A, and *eae*A), capsule synthesis (*rfc*), and toxins (*cnf*1, *hly*A, *elt*A, *est*A, *exh*A, *stx*1, and *stx*2) (Zhao et al., 2021). They divided *E. coli* into intestinal pathogenic *E. coli* (IPEC) and extraintestinal pathogenic *E. coli* (ExPEC) (Habouria et al., 2019). The IPEC is subclassified into enteropathogenic *E. coli* (EPEC), enterotoxigenic *E. coli* (ETEC), enteroaggregative *E. coli* (EAEC), enteroinvasive *E. coli* (EIEC), and enterohemorrhagic *E. coli* (EHEC). *pap*C is the most important subunit of the P fimbrial, as well as the *pap*A, which makes the P fimbrial of particular importance. Once connected, bacteria create biofilms under certain conditions, such as environmental stresses. In the event of biofilms attacking the mucous membrane of the intestine and contact with the epithelial cells of the intestine, a pathological situation is created (Mulder et al., 2011; Thanassi et al., 2012).

We exploited the fact that *K. pneumoniae* is highly pathogenic and investigated the virulence factors associated with *K. pneumoniae*. The *fim* gene and *mrl* gene clusters are the key virulence factors. *fim* genes are responsible for the production of type 1 pili (Potter et al., 2018). It is known that type 3 fimbriae belonging to the chaperone-usher class of fimbriae are encoded by five *mrk* genes (A, B, C, D, and F) (Ong et al., 2010). These two gene families support the concept that the *K. pneumoniae* isolates in this study may be highly virulent strains. *fep* genes were identified, which are documented to be required for catecholate siderophore translocation in the cytoplasm (Caza et al., 2015). Enterobactin (encoded by *ent* genes) is the strongest siderophore known which acquires iron for bacterial systems during pathogenesis. This iron uptake system that uses siderophores is one of the strategies employed by bacteria to increase its pathogenicity and is a determining factor in the outcome of infection (Mosbahi et al., 2018). *iucA*, *iucC*, and *iutA* genes identified on virulence plasmids encode aerobactin and its cognate receptor. Aerobactin is the most critical factor contributing to the high pathogenicity of hvKP. Clinically, aerobactin is much less prevalent (less than 10%) than other siderophores (Lam et al., 2018b) and is frequently produced by these isolates, causing liver abscesses, endophthalmitis, and other metastatic infections. Thus, hvKP strains are often defined based on aerobactin detection (Zhang et al., 2016; Liu and Guo, 2018). In addition, *rmpA* and *rmpA2* were detected in the SJ11 isolate. According to reports, the presence of *iuc* and *iro* is strongly associated with the presence of *rmpA*. *ybt* genes were identified in this study, supporting the evidence that these genes are associated with nosocomial infections. The *ybt* locus is mobilized in the *K. pneumoniae* population by an integrative, conjugative element termed ICEKp (virulence-associated integrative conjugative element of *K. pneumoniae*) and is commonly found among strains causing community-acquired infections as well as those causing healthcare-associated infections (Lam et al., 2018a).

IncF, IncA/IncC, and IncX are the most prevalent in carbapenemase production (Kopotsa et al., 2019). IncF is mostly associated with ESBLs, including carbapenemase genes (Peirano et al., 2017; Dong et al., 2018a,b; Gu et al., 2018; Yu et al., 2018; Domokos et al., 2019; Fu et al., 2019; Kim and Ko, 2019). Multiple plasmids have been reported, since 2012 and until recently, to carry *bla*_NDM_ variants and other determinants of aminoglycoside and tetracycline resistance, particularly on IncFIB and IncFII plasmid types in *K. pneumoniae* and *E. coli*, respectively (Domokos et al., 2019; Fu et al., 2019; Kim and Ko, 2019).

IncX3 is the predominant subgroup reported to harbor both *bla*_KPC_ and *bla*_NDM_. Further, *bla*_NDM–1_ and *bla*_NDM–5_ were more frequently associated with IncX3 than any other *bla*_NDM_ variant. Plasmid IncX3 related to the transmission of *bla*_NDM_ and *mcr-1* has also been found in most isolates. In our reports, IncX3 was detected in 11/17 *mcr-1*-positive *E. coli*. Although IncX3 plasmids are considered low-prevalence, narrow-host-range plasmids of Enterobacteriaceae, they have a wide range of host adaptability in *E. coli*. These plasmids may have served as a common vehicle in disseminating *bla*_NDM_ in *E. coli* among human, animal, and environment sectors and might be responsible for the rapid spread of NDM-carrying isolates.

There was no observed phylogenetic overlap in the distribution of *K. pneumoniae* between retail meat products and patients. Considering the MIC results, KPC-ST11 had higher prevalence of resistance to amikacin, aztreonam, and fosfomycin when compared with non-ST11 isolates, which corroborates recent reports on clinical isolates recovered from a hospital setting (Wang et al., 2018; Fu et al., 2019). Due to the high transmissibility of MDR, *K. pneumoniae* ST11 poses a serious threat to public health (Gu et al., 2018). Although there is currently no evidence that ST11 *K. pneumoniae* is transmitted from humans to animal-derived foods or animals, we need to monitor this potential vector for dissemination.

Our results also show that highly similar isolates have been previously identified from human samples, highlighting the importance of AMR surveillance and proper hygiene measures for meat products, indicating a possible transmission route of CR-harboring plasmids *via* meat sources. Although the use of carbapenems is regulated and forbidden in farms, the abuse of other beta-lactam antibiotics may result in the emergence of CRE, threatening the development of animal husbandry while attacking human health through direct connection with animal-derived foods. *E. coli* is an opportunistic pathogen, and *K. pneumoniae* is an important nosocomial pathogen. Diseases caused by *K. pneumoniae* account for more than 95% of *Klebsiella* infections (Lan et al., 2021). Therefore, the emergence of CRE in retail meat products suggests that CRE strains may spread between animals and humans.

## Conclusion

In summary, high numbers of the *bla*_NDM_-like genes and diversified plasmid types and STs were found. Isolates from the three sources are different in phylogeny, each having a main ST, but there are still some overlaps. The dissemination of *E. coli* and *K. pneumoniae* can be considered a clonal spread due to the similar STs. As repositories of ARGs, they have a great risk, which deserves more attention. Some isolates harbored transferable plasmids, in which many replicon types have been shown to be of important epidemiological significance. However, this study is limited to its sample type (especially with respect to porcine fecal samples), which could impact representativeness.

We conclude that with the growing prevalence of CRE being one of the major global threats for public health, the risk of zoonotic transmission *via* retail meat products is noteworthy. To further improve monitoring programs, more extensive and meticulous studies to analyze the phylogenetic relationships of different sources of CRE strains and transmission mechanisms of ARGs need to be developed.

## Data Availability Statement

The datasets presented in this study can be found in online repositories. The name of the repository and accession number can be found below: National Center for Biotechnology Information (NCBI) BioProject, https://www.ncbi.nlm.nih.gov/bioproject/, PRJNA624224.

## Ethics Statement

Ethical review and approval was not required for the animal study because retail meat products were collected from regular and qualified butcher shops. Written informed consent was obtained from the owners for the participation of their animals in this study. Ethical review and approval was not required for the study on human participants in accordance with the local legislation and institutional requirements. Written informed consent to participate in this study was provided by the participants’ legal guardian/next of kin.

## Author Contributions

JF, QX, ZY, LB, and JW conceived and designed the project. JF, QX, JM, and PZ performed the experiments. JF, QX, KL, MS, and KW analyzed the data. JF and QX wrote the manuscript. RL, DH, ZY, LB, and JW revised the manuscript. All authors contributed to the article and approved the submitted version.

## Conflict of Interest

The authors declare that the research was conducted in the absence of any commercial or financial relationships that could be construed as a potential conflict of interest.

## Publisher’s Note

All claims expressed in this article are solely those of the authors and do not necessarily represent those of their affiliated organizations, or those of the publisher, the editors and the reviewers. Any product that may be evaluated in this article, or claim that may be made by its manufacturer, is not guaranteed or endorsed by the publisher.

## Footnotes

1
https://www.mic.eucast.org/Eucast2/

2
https://rast.nmpdr.org/

3
https://cge.cbs.dtu.dk/services/ResFinder/

4
https://cge.cbs.dtu.dk//services/PlasmidFinder/

5
http://bacdb.cn/BacWGSTdb/index.php

6
https://itol.embl.de/

7
http://www.mgc.ac.cn/VFs/

8
https://doi.org/10.1093/nar/gki008

9
https://www.ncbi.nlm.nih.gov/bioproject
