# Supplementary material for: Characterization of Carbapenem-Resistant Enterobacteriaceae Cultured From Retail Meat Products, Patients, and Porcine Excrement in China
Source: Front Microbiol. 2021 Dec 23;12:743468. doi: 10.3389/fmicb.2021.743468 (PMC8734966; doi:10.3389/fmicb.2021.743468)
Supplement: Supplementary file 1 [file Data_Sheet_1.docx]

**Supplementary files:**

**Table S1** Antimicrobial agent list

| Antimicrobial agent | | MIC breakpoints (µg/mL) | | |
| --- | --- | --- | --- | --- |
|  |  | S | I | R |
| Ampicillin | AMP | ≤8 | 16 | ≥32 |
| Cefotaxime | CTX | ≤1 | 2 | ≥4 |
| Cefepime | FEP | ≤2 | 4-8 | ≥16 |
| Ceftazidime | CAZ | ≤4 | 8 | ≥16 |
| Imipenem | IMI | ≤1 | 2 | ≥4 |
| Meropenem | MEM | ≤1 | 2 | ≥4 |
| Gentamicin | GEN | ≤4 | 8 | ≥16 |
| Amikacin | AMI | ≤16 | 32 | ≥64 |
| Streptomycin | STR | - | - | - |
| Sulfonamides | Sul | ≤256 | - | ≥512 |
| Trimethoprim-sulfamethoxazole | SXT | ≤0.12 | 0.25 | ≥0.5 |
| Chloramphenicol | CHL | ≤8 | 16 | ≥32 |
| Azithromycin | AZI | ≤16 | - | ≥32 |
| Tetracycline | TET | ≤4 | 8 | ≥16 |
| Nalidixic acid | NAL | ≤16 | - | ≥32 |
| Ciprofloxacin | CIP | ≤0.25 | 0.5 | ≥1 |
| Polymyxin B | PB | - | ≤2 | ≥4 |

**Table S2**Source of isolations

| **source** | **species** | **amount** |
| --- | --- | --- |
| **Retail meat products** | *E. coli* | 30 |
|  | *K. pneumoniae* | 5 |
|  | *E. cloacae* | 1 |
| **Porcine excrements** | *E. coli* | 5 |
| **Patients** | *E. coli* | 8 |
|  | *K. pneumoniae* | 4 |

**Table S3** Acquired antimicrobial resistance genes of the 53 CREs

| Strain | Species | Acquired antimicrobial resistance gene-Results | | | | | | | | | | | |
| --- | --- | --- | --- | --- | --- | --- | --- | --- | --- | --- | --- | --- | --- |
|  |  | Aminoglycoside | Beta-lactam | Colistin | Fluoroquinolone | Fosfomycin | Macrolides | [Quinolones](javascript:;) | Phenicol | Rifampicin | Sulphonamide | Tetracycline | Trimethoprim |
| HK14 | *K. pneumoniae* | aph(3'')-lb, aac(6')-lb-cr, aph(6)-ld, aph(3')-la, aac(6')-ll | CTX-M-15, CMY-2, TEM-1B, SHV-28, NDM-9, OXA-1 |  | aac(6')-lb-cr, oqxA, oqxB, qnrB1 | fosA |  |  | catB3, floR |  | sul1, sul2 | tet(A) | dfrA14 |
| HK19 | *K. pneumoniae* | aph(3')-la | CTX-M-65, SHV-182, KPC-2 |  | oqxA, oqxB | fosA | mph(A) |  | catA2 |  | sul1 | tet(A) | dfrA1 |
| SJ11 | *K. pneumoniae* | aadA2, rmtB | TEM-1B, KPC-2, SHV-12, CTX-M-65 |  | oqxA, oqxB | fosA |  |  |  |  | sul1 |  |  |
| SJ13 | *K. pneumoniae* | aadA16, aac(6')-lb-cr, aph(3')-la, aac(3)-lld, aph(6)-ld, aph(3'')-lb | NDM-7, OKP-A-5 |  | aac(6')-lb-cr, oqxA, oqxB, qnrB52 | fosA | mph(A) |  | floR | ARR-3 | sul1, sul1 | tet(A) | dfrA27 |
| SJ17 | *K. pneumoniae* |  | CTX-M-65, SHV-182, KPC-2 |  | oqxA, oqxB | fosA | mph(A) |  | catA2 |  | sul1 | tet(A) | dfrA1 |
| SJ18 | *K. pneumoniae* | aadA2, rmtB | TEM-1B, KPC-2, SHV-12, CTX-M-65 |  | oqxA, oqxB | fosA |  |  |  |  | sul1 |  |  |
| SJ24 | *K. pneumoniae* | aac(3)-lld, aph(3')-la | TEM-1B, KPC-2, SHV-182, CTX-M-14, NDM-5 |  | oqxA, oqxB | fosA | mdf(A), mph(A) |  | catA2 |  | sul1 | tet(A) | dfrA1 |
| SJ32 | *K. pneumoniae* | aadA16, aac(6')-lb-cr, aph(3')-la, aac(3)-lld, aph(4)-la, aph(6)-ld, aph(3'')-lb | CTX-M-27, SHV-182, NDM-5 |  | aac(6')-lb-cr, qnrB2, oqxA, oqxB | fosA | mph(A) |  |  | ARR-3 | sul1, sul1, sul2 | tet(A) | dfrA27 |
| SJ9 | *K. pneumoniae* | aadA16, aac(6')-lb-cr, aph(3')-la, aac(3)-lld, aph(6)-ld, aph(3'')-lb | TEM-1B, SHV-81, NDM-5, CTX-M-3 |  | aac(6')-lb-cr, qnrS1, oqxA, oqxB, qnrB2 | fosA | mph(A) |  | floR | ARR-3 | sul1, sul1, sul2 | tet(A) | dfrA27 |
| SJE44 | *E. cloacae* | aph(4)-la, aac(3)-lV, aph(3')-la, aac(6')-lb-cr | NDM-1, CMH-3, OXA-1, DHA-1 |  | qnrB4, aac(6')-lb-cr | fosA | mph(A) |  | catB3, floR | ARR-3 | sul1, sul1, sul2 | tet(D) |  |
| HK1 | *E. coli* | aadA5, aph(6)-ld, aac(3)-lld, aph(3'')-lb | blaCTX-M-55, blaNDM-5 | - | - | fosA3 | mph(A), mdf(A) |  |  |  | sul1, sul2 | tet(A) | dfrA17 |
| HK11 | *E. coli* | aph(3'')-lb, aph(4)-la, aac(3)-IV, aadA15, aac(6')-lb3, aph(6)-ld, aph(3')-la, aac(6')-lla, armA, aadA1 | CTX-M-14, NDM-5 |  | aac(6')-lb-cr | fosA3 | mdf(A), msr(E), mph€ |  | floR, cmlA1 |  | sul1, sul2, sul3 | tet(A) | dfrA1 |
| HK13 | *E. coli* | aph(3'')-lb, aph(4)-la, aac(3)-IV, aph(6)-ld, aph(3')-la, aadA1 | CTX-M-14, NDM-5 | mcr-1.1 | oqxA, oqxB | fosA3 | mdf(A) |  | catA2, cmlA1, floR |  | sul1, sul3 | tet(A) | dfrA12 |
| HK15 | *E. coli* | aac(3)-lla, rmtB, aph(3'')-lb, aph(6)-ld | CTX-M-64, NDM-5 |  |  | fosA3 | mph(A), mdf(A) |  | floR |  | sul2 | tet(A) | dfrA14 |
| HK16 | *E. coli* | aph(3'')-lb, aph(4)-la, aac(3)-IV, aph(6)-ld, aph(3')-la, aadA1 | CTX-M-14, NDM-5 | mcr-1.1 | oqxA, oqxB | fosA3 | mdf(A) |  | catA2, cmlA1, floR |  | sul1, sul3 | tet(A) | dfrA12 |
| HK17 | *E. coli* | aph(3')-lla, aadA2, aph(3'')-lb, aac(3)-lld, aph(6)-ld, aac(6')-lb-cr, aadA1, aadA5 | TEM-1B, OXA-1, NDM-5 |  | aac(6')-lb-cr, qnrS1, qnrS2, oqxA, oqxB |  | mdf(A) |  | catB3, floR, cmlA1 | ARR-3 | sul2, sul3 | tet(A) | dfrA17 |
| HK18 | *E. coli* | aadA2, aph(3')-la, aph(3'')-lb, aph(6)-ld | CTX-M-14, NDM-5 | mcr-1.1 | oqxA, oqxB | fosA3 | mph(A), mdf(A) |  | floR |  | sul1, sul2 | tet(A) | dfrA12 |
| HK2 | *E. coli* | aph(3'')-lb, aadA2, aadA5, aph(3')-la, aadA1, aph(4)-la, aac(3)-IV, aph(6)-ld | NDM-5, CTX-M-14, TEM-1B, CARB-2 | mcr-1.1 |  | fosA3 | mph(A), mdf(A) |  | floR, cmlA1 |  | sul1, sul3 | tet(M), tet(A) | dfrA17, dfrA16 |
| HK20 | *E. coli* | aph(3')-la, aph(3'')-lb, aadA2, aph(6)-ld, aac(3)-lld | TEM-1B, NDM-5 | mcr-1.1 | oqxA, oqxB | fosA3 | mph(A), mdf(A) |  | floR |  | sul2 | tet(A) | dfrA12 |
| HK4 | *E. coli* | rmtB, aac(6')-lb-cr, aph(4)-la, aph(3')-la, aac(3)-IV | NDM-9, TEM-1B, OXA-1 |  | aac(6')-lb-cr | fosA3 | mdf(A), mph(A), |  | catB3, cmlA1, floR | ARR-3 | sul1, sul2, sul3 | tet(A) | dfrA12 |
| HK5 | *E. coli* | aph(3'')-lb, aac(3)-lla, aph(6)-ld, aph(3')-la | CTX-M-55, NDM-5 |  |  |  | mdf(A), | lnu(F) | floR |  | sul2, sul3 | tet(A) |  |
| HK9 | *E. coli* | aadA5, aph(3')-la, aph(6)-ld, aph(3'')-lb | TEM-1B, NDM-5 |  |  |  | mdf(A), mph(A) |  | floR |  | sul1, sul2 | tet(A) | dfrA17 |
| SJ1 | *E. coli* | aadA5, aac(6')-lb3, aac(3)-lld | TEM-1B, CTX-M-14, NDM-5 |  | aac(6')-lb-cr |  | mdf(A), mph(A), |  | cmlA1 |  | sul1 | tet(B) | dfrA17 |
| SJ10 | *E. coli* | aac(3)-lla, aph(3')-la, aadA2 | TEM-1B, CTX-M-55, NDM-5 | mcr-1.1 |  | fosA3 | mph(A), mdf(A) | , lnu(F) | floR |  | sul1, sul3 | tet(A) | dfrA12 |
| SJ12 | *E. coli* | aph(3')-la, aac(3)-lld, aadA5 | TEM-1B, CTX-M-14, NDM-5 |  |  |  | mph(A), mdf(A) |  | catA1 |  | sul1 | tet(B) | dfrA17 |
| SJ15 | *E. coli* | aph(3')-la, aac(3)-lld, aadA5 | TEM-1B, CTX-M-14, NDM-5 |  |  |  | mph(A), mdf(A) |  | catA1 |  | sul1 | tet(B) | dfrA17 |
| SJ16 | *E. coli* | aph(3'')-lb, aac(6')-lb-cr, aph(6)-ld, aadA16, aac(3)-lld, rmtB | TEM-1B, NDM-5 |  | aac(6')-lb-cr |  | mph(A), mdf(A) |  | catA1, floR | ARR-3 | sul1, sul2 | tet(A) | dfrA14, dfrA27 |
| SJ19 | *E. coli* | aph(4)-la, aac(3)-IV, aph(3')-la | CTX-M-14, NDM-5 | mcr-1.1 | oqxA, oqxB | fosA3 | mdf(A), mph(A) |  | cmlA1, floR |  | sul1, sul2, sul3 | tet(A), tet(M) | dfrA12 |
| SJ2 | *E. coli* | aac(3)-lld, aadA2, aadA1, aph(3')-la, aph(3'')-lb, aph(6)-ld | CTX-M-14, NDM-5 |  |  | fosA3 | mdf(A), mph(A) |  | floR |  | sul1, sul2 | tet(A) | dfrA1, dfrA12 |
| SJ20 | *E. coli* | aph(3')-la, aac(3)-lld, aadA5 | CTX-M-14, NDM-5, TEM-1B |  |  |  | mdf(A), mph(A) |  | catA1 |  | sul1 | tet(B) | dfrA17 |
| SJ21 | *E. coli* | aac(6')-lb-cr, aph(4)-la, aac(3)-lV, aadA16 | CTX-M-65, NDM-5, OXA-1 | mcr-1.1 | aac(6')-lb-cr, oqxA, oqxB |  | mdf(A), mph(A) |  | catB3, floR |  | sul1, sul2 | tet(A) | dfrA27 |
| SJ22 | *E. coli* | aac(3)-lld, aadA8b | NDM-5, TEM-1B | mcr-1.1 | qnrS2 |  | mdf(A), mph(A) |  |  |  | sul2, sul3 | tet(A), tet(M) | dfrA12 |
| SJ23 | *E. coli* | aac(6')-lb3, aac(3)-lla, aph(3')-la, aadA5 | CTX-M-55, NDM-5 | mcr-1.1 | aac(6')-lb-cr, oqxA, oqxB | fosA3 | , mdf(A) | lnu(F) | floR |  | sul3 | tet(A) | dfrA17 |
| SJ25 | *E. coli* | rmtB, aph(3'')-lb, aac(6')-lb-cr, aph(6)-ld, aadA2, aac(3)-lld | CTX-M-15, NDM-5, TEM-1B, OXA-1 |  | aac(6')-lb-cr, qnrS1 |  | mdf(A), mph(A) |  | catB3 |  | sul1, sul2 | tet(A) | dfrA12 |
| SJ26 | *E. coli* | rmtB, aph(3'')-lb, aph(6)-ld, aac(3)-lld | NDM-5, TEM-1B |  | qnrS1 |  | mdf(A), mph(A) |  |  |  | sul1, sul2 | tet(A) |  |
| SJ27 | *E. coli* | rmtB, aph(3'')-lb, aac(6')-lb-cr, aph(6)-ld, aadA2, aac(3)-lld | CTX-M-15, NDM-5, TEM-1B, OXA-1 |  | aac(6')-lb-cr, qnrS1 |  | mdf(A), mph(A) |  | catB3 |  | sul1, sul2 | tet(A) | dfrA12 |
| SJ28 | *E. coli* | rmtB, aph(3'')-lb, aac(6')-lb-cr, aph(6)-ld, aadA2, aac(3)-lld | CTX-M-15, NDM-5, TEM-1B, OXA-1 |  | aac(6')-lb-cr, qnrS1 |  | mdf(A), mph(A) |  | catB3 |  | sul1, sul2 | tet(A) | dfrA12 |
| SJ29 | *E. coli* | aadA16, aph(3')-la, aadA22, aph(3'')-lb, aph(6)-ld | CTX-M-55, NDM-1, TEM-1B | mcr-1.1 |  |  | mdf(A), mph(A), |  | catB3, floR |  | sul1, sul2 | tet(A), tet(M) | dfrA27 |
| SJ3 | *E. coli* | aph(3')-la, aac(3)-lld, aadA5 | TEM-1B, CTX-M-14, NDM-5 |  |  |  | mdf(A), mph(A) |  | catA1 |  | sul1 | tet(B) | dfrA17 |
| SJ30 | *E. coli* | rmtB, aph(3'')-lb, aadA5, aph(6)-ld, aadA2 | CTX-M-55, NDM-9, TEM-1B | mcr-1.1 | qepA1 | fosA3 | mph(A), mdf(A) |  | floR, catA1 |  | sul1, sul2 | tet(A) | dfrA12, dfrA17 |
| SJ31 | *E. coli* | aph(3'')-lb, aph(4)-la, aac(3)-lV, aadA15, aac(6')-lb3, aph(6)-ld, aac(6')-lla, armA, aadA1 | CTX-M-14, NDM-5 |  | aac(6')-lb-cr | fosA3 | mdf(A), msr(E), mph(E) | | floR, cmlA1 |  | sul1, sul2, sul3 | tet(A) | dfrA1 |
| SJ34 | *E. coli* | aac(6')-lb3, aac(3)-lla, aph(3')-la, aadA5 | CTX-M-15, NDM-5 | mcr-1.1 | aac(6')-lb-cr, oqxA, oqxB | fosA3 | mdf(A) | lnu(F), | floR |  | sul1, sul3 | tet(A) | dfrA17 |
| SJ36 | *E. coli* | rmtB, aph(3'')-lb, aac(6')-lb-cr, aph(3')-la, aph(6)-ld, aadA2, aac(3)-lld | CTX-M-15, NDM-5, TEM-1B, OXA-1 |  | aac(6')-lb-cr, qnrS1 |  | mdf(A), mph(A) |  | catB3 |  | sul1, sul2 | tet(A) | dfrA12 |
| SJ4 | *E. coli* | aph(3')-la, aph(3'')-lb, aph(6)-ld, aadA2, aadA22 | CTX-M-14, NDM-9, TEM-1B | mcr-1.1 |  | fosA3 | mph(A), mdf(A), |  | floR, cml |  | sul1, sul2 | tet(A) | dfrA12 |
| SJ6 | *E. coli* | aac(6')-lb3, aac(3)-lla, aph(3')-la, aadA5 | CTX-M-55, NDM-5 | mcr-1.1 | aac(6')-lb-cr, oqxA, oqxB | fosA3 | mdf(A) | lnu(F), | floR |  | sul3 | tet(A) | dfrA17 |
| SJ7 | *E. coli* | aph(3')-la, aac(3)-lld, aadA5 | CTX-M-14, NDM-5, TEM-1B |  |  |  | mdf(A), mph(A) |  | catA1 |  | sul1 | tet(B) | dfrA17 |
| SJE39 | *E. coli* | aph(3')-la, aadA2 | NDM-5, TEM-1B | mcr-1.1 | oqxA, oqxB |  | mdf(A) |  | cmlA1, floR |  | sul1, sul3 | tet(A) |  |
| SK1 | *E. coli* | aadA1, aph(3')-la, aac(3)-lld | NDM-5, TEM-1B |  |  |  | mdf(A) | lnu(F), | cmlA1, floR |  | sul2, sul3 | tet(A), tet(M) | dfrA12 |
| SK2 | *E. coli* | aadA1, aph(3')-la, aac(3)-lld | NDM-5, TEM-1B | mcr-1.1 |  |  | mdf(A) | lnu(F), | cmlA1, floR |  | sul2, sul3 | tet(A), tet(M) | dfrA12 |
| SK3 | *E. coli* | aadA1, aph(3')-la, aac(3)-lld | NDM-5, TEM-1B |  |  |  | mdf(A) | lnu(F), | cmlA1, floR |  | sul2, sul3 | tet(A), tet(M) | dfrA12 |
| SK4 | *E. coli* | aph(3')-la, aac(3)-lld, aadA5 | CTX-M-14, NDM-5, TEM-1B |  |  |  | mdf(A), mph(A) |  | catA1 |  | sul1 | tet(B) | dfrA17 |
| SK5 | *E. coli* | aadA1, aph(3')-la, aac(3)-lld | NDM-5, TEM-1B |  |  |  | mdf(A) | lnu(F), | cmlA1, floR |  | sul2, sul3 | tet(A), tet(M) | dfrA12 |
| SK7 | *E. coli* |  | NDM-5 |  |  |  | mdf(A) |  |  |  |  | tet(A), tetB(P), tetA(P) |  |

**Table S4** virulence genes of *E. coli* and *K. pneumoniae* strains in this study

| strain | species | source | virulence genes |
| --- | --- | --- | --- |
| SJ2 | *E. coli* | retail meat products | air, cea, chuA, eilA, gad, hra, ireA, iucC, iutA, kpsE, kpsM, lpfA, sitA, terC, traT, usp, yfcV |
| SJ3 | *E. coli* | retail meat products | cea, cib, cvaC, etsC, gad, hlyF, iroN, iss, iucC, iutA, mchF, ompT, terC, traT |
| SJ4 | *E. coli* | retail meat products | cea, celb, cma, cvaC, hlyF, hra, iroN, iss, iucC, iutA, lpfA, ompT, papA, sitA, terC, traT |
| SJ6 | *E. coli* | retail meat products | astA, cma, cvaC, gad, hlyF, hra, iss, iucC, iutA, lpfA, ompT,papC, sitA, terC, traT |
| SJ7 | *E. coli* | retail meat products | cea, cib, cvaC, etsC, gad, hlyF, iroN, iss, iucC, iutA, mchF, ompT, terC, traT |
| SJ10 | *E. coli* | retail meat products | astA, cma, cvaC, gad, hlyF, hra, iss, iucC, iutA, lpfA, ompT,papC, sitA, terC, traT |
| SJ12 | *E. coli* | retail meat products | cea, cib, cvaC, etsC, gad, hlyF, iroN, iss, iucC, iutA, mchF, ompT, terC, traT |
| SJ15 | *E. coli* | retail meat products | cea, cib, cvaC, etsC, gad, hlyF, iroN, iss, iucC, iutA, mchF, ompT, sitA, terC, traT |
| SJ19 | *E. coli* | retail meat products | gad, terC |
| SJ20 | *E. coli* | retail meat products | cea, cib, cvaC, etsC, gad, hlyF, iroN, iss, iucC, iutA, mchF, ompT, sitA, terC, traT |
| SJ21 | *E. coli* | retail meat products | astA, gad, hra, terC |
| SJ22 | *E. coli* | retail meat products | gad, iss, iucC, iutA, lpfA, ompT, sitA, terC, traT |
| SJ23 | *E. coli* | retail meat products | astA, cma, cvaC, gad, hlyF, hra, iss, iucC, iutA, lpfA, ompT,papC, sitA, terC, traT |
| SJ29 | *E. coli* | retail meat products | air, astA, cea, chuA, cia, eilA, etsC, gad, ibeA, iha, iucC, iutA, kpsE, kpsMII, lpfA, sitA, terC, traT, usp, yfcV |
| SJ30 | *E. coli* | retail meat products | astA, cba, cia, cma, etsC ,gad, hlyF, hra, iss, iucC, iutA, lpfA, ompT, papA, sitA, terC, traT, tsh |
| SJ34 | *E. coli* | retail meat products | astA, cma, cvaC, gad, hlyF, hra, iss, iucC, iutA, lpfA, ompT,papC, sitA, terC, traT |
| SJ36 | *E. coli* | retail meat products | capU, gad, hra, iss, terC |
| SJE39 | *E. coli* | retail meat products | gad, terC |
| HK1 | *E. coli* | retail meat products | gad, hlyF, iss, neuC, ompT, sitA, terC, traT |
| HK2 | *E. coli* | retail meat products | terC |
| HK4 | *E. coli* | retail meat products | air, chuA, cma, cvaC, eilA, gad, hlyF, hra, iroN, iss, iucC, iutA, ompT, sitA, terC, traT |
| HK5 | *E. coli* | retail meat products | air, chuA, cma, cvaC, eilA, gad, hlyF, hra, ibeA, irp2, iucC, iutA, kpsE, kpsM, lpfA, mcmA, ompT, sitA, terC, traT, usp, yfcV |
| HK9 | *E. coli* | retail meat products | astA, etsC, fyuA, gad, irp2, sitA, terC, traT |
| HK11 | *E. coli* | retail meat products | air, chuA, cma, cvaC, eilA, fyuA, hlyF, hra, ibeA, irp2, iucC, iutA, kpsE, kpsM, lpfA, mcmA, ompT, papC, sitA, terC, traT, usp, yfcV |
| HK13 | *E. coli* | retail meat products | chuA, cma, cvaC, eilA, fyuA, hlyF, hra, ibeA, irp2, iucC, iutA, kpsE, kpsM, lpfA, mcmA, ompT, papC, sitA, terC, traT, usp, yfcV |
| HK15 | *E. coli* | retail meat products | air, chuA, eilA, hlyF, hra, iss, kpsE, kpsMII, lpfA, ompT, papA, sitA, terC, traT, usp, yfcV |
| HK16 | *E. coli* | retail meat products | air, chuA, cma, cvaC, eilA, fyuA, hlyF, hra, ibeA, irp2, iucC, iutA, kpsE, kpsM, lpfA, mcmA, ompT, papC, sitA, terC, traT, usp, yfcV |
| HK17 | *E. coli* | retail meat products | astA, gad, hra, terC |
| HK18 | *E. coli* | retail meat products | astA, gad, iss, lpfA, terC |
| HK20 | *E. coli* | retail meat products | astA, cma, cvaC, gad, hlyF, iroN, iss, iucC, iutA, lpfA, ompT,papC, sitA, terC, traT |
| SJ1 | *E. coli* | patients | capU, gad, iss, terC, traT |
| SJ16 | *E. coli* | patients | astA, gad, iss, lpfA, ompT, papC, terC, traT, yfcV |
| SJ25 | *E. coli* | patients | capU, gad, hra, iss, terC |
| SJ26 | *E. coli* | patients | capU, gad, hra, iss, terC |
| SJ27 | *E. coli* | patients | capU, gad, hra, iss, terC |
| SJ28 | *E. coli* | patients | capU, gad, hra, iss, terC |
| SJ31 | *E. coli* | patients | air, chuA, cma, cvaC, eilA, fyuA, gad, hlyF, hra, ibeA, irp2, iucC, iutA, kpsE, kpsM, lpfA, mcmA, ompT, papC, sitA, terC, traT, usp, yfcV |
| SK1 | *E. coli* | porcine excrements | terC, traT |
| SK2 | *E. coli* | porcine excrements | gad, terC, traT |
| SK3 | *E. coli* | porcine excrements | gad, terC, traT |
| SK4 | *E. coli* | porcine excrements | cea, cib, cvaC, etsC, gad, hlyF, iroN, iss, iucC, iutA, mchF, ompT, sitA, terC, traT |
| SK5 | *E. coli* | porcine excrements | gad, terC, traT |
| SK7 | *E. coli* | porcine excrements | gad, ompT, terC |
| SJ9 | *K. pneumoniae* | retail meat products | clpV/tssH, dotU/tssL, entA, entB, entC, entD, entE, entF, entS, fepA, fepB, fepC, fepD, fepG, fimA, fimB, fimC, fimD, fimE, fimF, fimG, fimH, fimI, fimK, hcp/tssD, icmF/tssM, impA/tssA, mrkA, mrkB, mrkC,mrkD, mrkF, mrkH, mrkI, mrkJ, sciN/tssJ, tssF, tssG, vasE/tssK, vgrG/tssI, vipA/tssB, vipB/tssC, acrA, acrB, clpV, dotU, fes, icmF, impA, impF, impG, impH, impJ, iroE, iroN, ompA, pilW, rcsA, rcsB, sciN, vgrG |
| SJ13 | *K. pneumoniae* | retail meat products | clpV/tssH, dotU/tssL, entA, entB, entC, entD, entE, entF, entS, fepA, fepB, fepC, fepD, fepG, fimA, fimB, fimC, fimD, fimE, fimF, fimG, fimH, fimI, hcp/tssD, icmF/tssM, mrkA, mrkB, mrkC, mrkD, mrkF, mrkH, mrkI, mrkJ, sciN/tssJ, tssF, tssG, vasE/tssK, vgrG/tssI, vipA/tssB, vipB/tssC, acrA, acrB, clpV, dotU, fes, icmF, impA, impF, impG, impH, impJ, iroE, ompA, pilW, rcsA, rcsB, sciN, vgrG |
| SJ32 | *K. pneumoniae* | retail meat products | clpV/tssH, dotU/tssL, entA, entB, entC, entD, entE, entF, entS, fepA, fepB, fepC, fepD, fepG, fimA, fimB, fimC, fimD, fimE, fimF, imG, fimH, fimI, fimK, hcp/tssD, icmF/tssM, impA/tssA, mrkA, mrkB, mrkC, mrkD, mrkF, mrkH, mrkI, mrkJ, sciN/tssJ, tssF, tssG, vasE/tssK, vgrG/tssI, vipA/tssB, vipB/tssC, acrA, acrB, clpV, dotU, fes, icmF, impA, impF, impG, impH, mpJ, iroE, iroN, ompA, rcsA, rcsB, sciN, tli1, vgrG |
| HK14 | *K. pneumoniae* | retail meat products | clpV/tssH, dotU/tssL, entA, entB, entC, entD, entE, entF, entS, fepA, fepB, fepC, fepD, fepG, fimA, fimB, fimC, fimD, fimE, fimF, fimG, fimH, fimI, fimK, hcp/tssD, icmF/tssM, impA/tssA, mrkA, mrkB,mrkC, mrkD, mrkF, mrkH, mrkI, mrkJ, sciN/tssJ, tssF, tssG, vasE/tssK, vgrG/tssI, acrA, acrB, fes, icmF, impA, impF, impG, impH, impJ, iroE, iroN, ompA, rcsA, rcsB, sciN, tli1 |
| SJ11 | *K. pneumoniae* | patients | clpV/tssH, dotU/tssL, entA, entB, entC, entD, entE, entF, entS, fepA, fepB, fepC, fepD, fepG, fimA, fimB, fimC, fimD, fimE, fimF, fimG, fimH, fimI, fimK, hcp/tssD, icmF/tssM, impA/tssA, mrkA, mrkB, mrkC, mrkD, mrkF, mrkH, mrkI, mrkJ, sciN/tssJ, tssF, tssG, vasE/tssK, vgrG/tssI, vipA/tssB, vipB/tssC, ybtA, ybtE, ybtP, ybtQ, ybtS, ybtT, ybtU, ybtX, acrA, acrB, clpV, dotU, fes, fyuA, impA, impF, impG, impH, impJ, iroE, iroN, irp1, irp2, ompA, pilW, rcsA, rcsB, rmpA, mpA2, sciN, tli1 |
| SJ17 | *K. pneumoniae* | patients | clpV/tssH, dotU/tssL, entA, entB, entC, entD, entE, entF, entS, fepA, fepB, fepC, fepD, fepG, fimA, fimB, fimC, fimD, fimE, fimF, fimG, fimH, fimI, fimK, hcp/tssD, icmF/tssM, impA/tssA , mrkA, mrkB, mrkC, mrkD, mrkF, mrkH, mrkI, mrkJ, sciN/tssJ, tssF, tssG, vasE/tssK, vgrG/tssI, vipA/tssB, vipB/tssC, ybtA, ybtE, ybtP, ybtQ, ybtS, ybtT, ybtU, ybtX, acrA, acrB, fes, fyuA, impA, impF, impG, impH, impJ , iroE, iroN, irp1, irp2, ompA, pilW, rcsA,rcsB, sciN, tli1, virB |
| SJ18 | *K. pneumoniae* | patients | clpV/tssH, dotU/tssL, entA, entB, entC, entD, entE, entF, entS, fepA, fepB, fepC, fepD, fepG, fimA, fimB, fimC, fimD, fimE, fimF, fimG, fimH, fimI, fimK, hcp/tssD, icmF/tssM, impA/tssA, mrkA, mrkB, rkC, mrkD, mrkF, mrkH, mrkI, mrkJ, sciN/tssJ, tssF, tssG, vasE/tssK, vgrG/tssI, vipA/tssB, vipB/tssC, ybtA, ybtE, ybtP, ybtQ, ybtS, ybtT, ybtU, ybtX, acrA, acrB, clpV, dotU, fes, fyuA, impA, impF, impG, impH, impJ, iroE, iroN, irp1, irp2, ompA, pilW rcsA, rcsB, sciN, tli1 |
| SJ24 | *K. pneumoniae* | patients | fimA, fimB, fimC, fimD, fimE, fimF, fimG, fimH, fimI, fimK, hcp/tssD, icmF/tssM, impA/tssA, mrkA, mrkB, mrkC, mrkD,mrkF, mrkH, mrkI, mrkJ, sciN/tssJ, tssF, tssG, vasE/tssK, vgrG/tssI, vipA/tssB, vipB/tssC, ybtA, ybtE, ybtP, ybtQ, ybtS, ybtT, ybtU, ybtX, acrA, acrB, aec16, clpV, dotU, ecpE, exeG, fes, flgI, flgK, fliE, fliH, fliM, fliN, fliR, fliS, fyuA, gspI, gspJ, hemG, icmF, impA, impF, impG, impH, impJ, iroD, iroE, iroN, irp1, irp2, ompA, pilT, pilV, pilW, rcsA, rcsB, sciN, sitC, tli1, vasA/impG, virB |
| HK19 | *K. pneumoniae* | patients | clpV/tssH, dotU/tssL, entA, entB, entC, entD, entE, entF, entS, fepA, fepB, fepC, fepD, fepG, fimA, fimB, fimC, fimD, fimE, fimF, fimG, fimH, fimI, fimK, hcp/tssD, icmF/tssM, impA/tssA, mrkA, mrkB, mrkC, mrkD, mrkF, mrkH, mrkI, mrkJ, sciN/tssJ, tssF, tssG, vasE/tssK, vgrG/tssI, vipA/tssB, vipB/tssC, ybtA, ybtE, ybtP, ybtQ, ybtS, ybtT, ybtU, ybtX, acrA, acrB, clpV, dotU, fes, fyuA, icmF, impA, impF, impG, impH, impJ, iroE, iroN, irp1, irp2, ompA, pilW, rcsA, rcsB, sciN, tli1, virB |


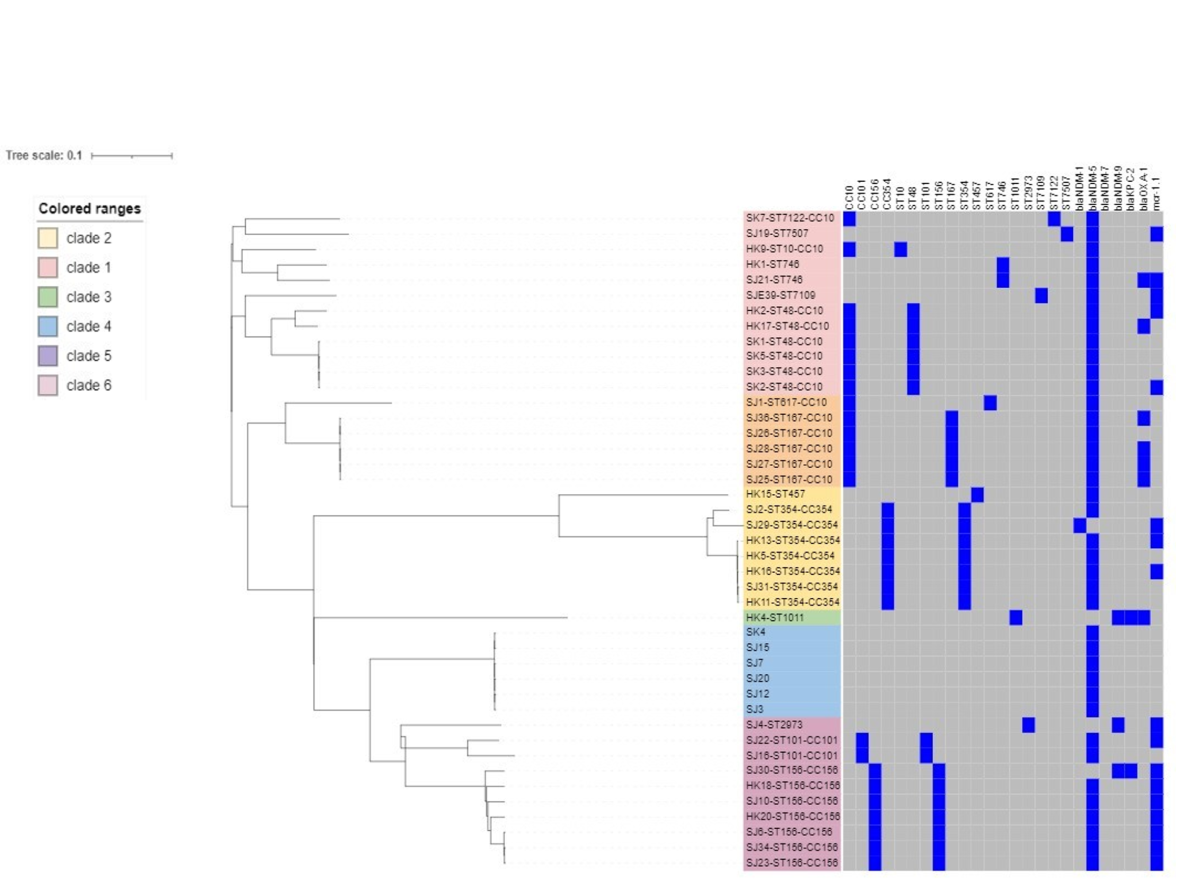


**Figure S1. Phylogenetic structures of the NDM-positive *E. coli* isolates from this study.** The maximum likelihood tree showsthe relationships among the 43 NDM-positive *E. coli*isolates and their CC types, STs, carbapenemase-genes and *mcr-1* gene.


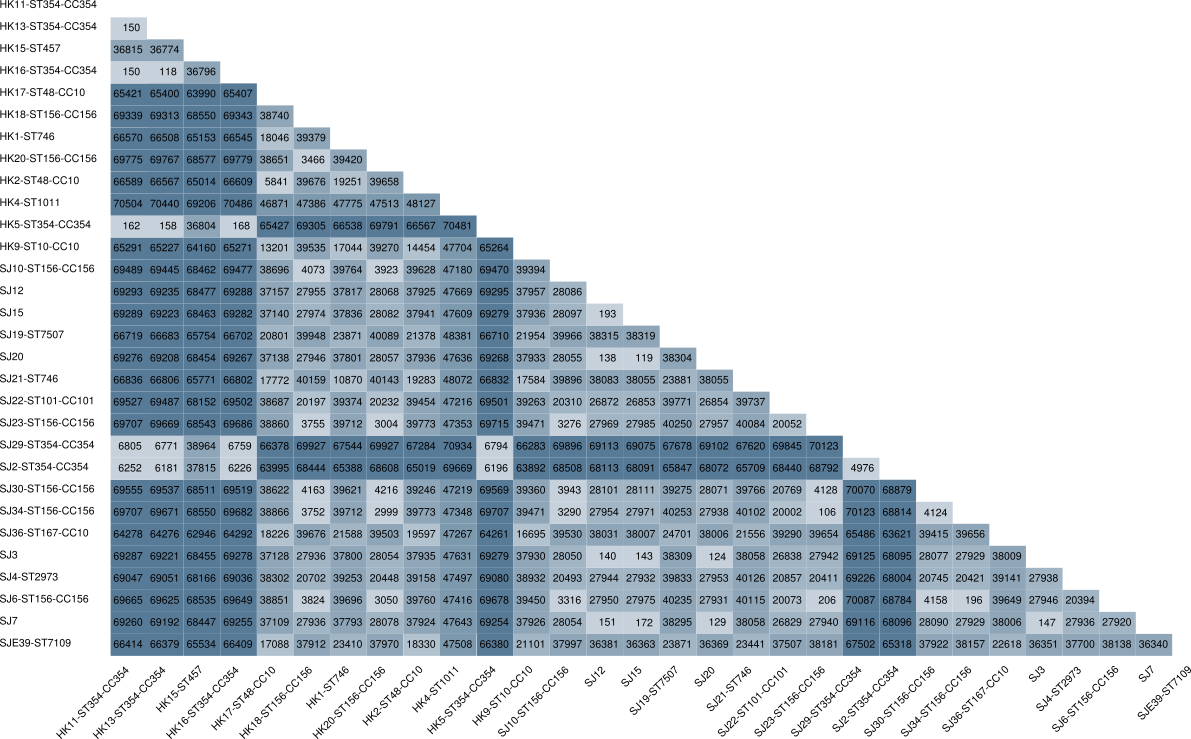


**Figure S2. Matrix of SNP pair counts among NDM-positive *E. coli* strains.** Number of SNP was calculated by comparing the genome sequences to the reference (MG1655) genome. Background colors represent different number of pairwise SNPs, ranging from a minimum of 118 (white background) to a maximum of 70504 (ultramarine).


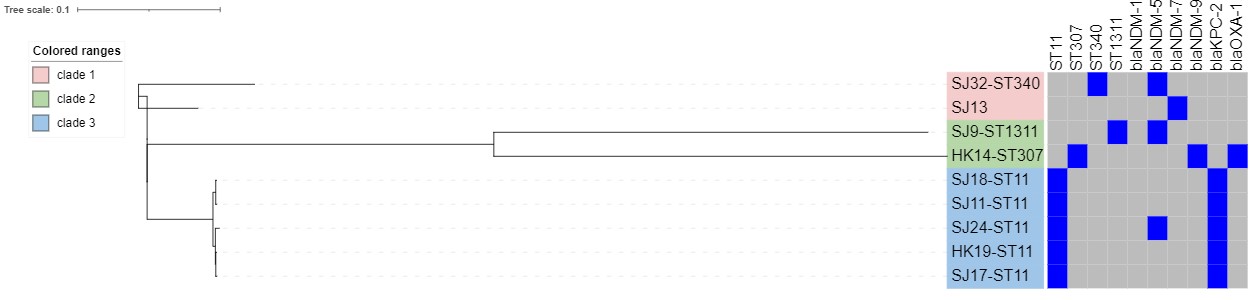


**Figure S3. Phylogenetic structures of the NDM-positive *K. pneumoniae* isolates from this study.** The maximum likelihood tree showsthe relationships among the 9 NDM-positive *K. pneumoniae* isolates and their STs and carbapenemase-genes.


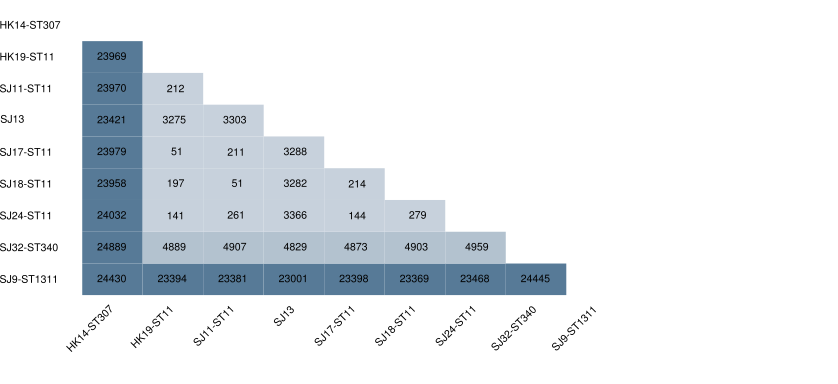


**Figure S4. Matrix of SNP pair counts among NDM-positive *K. pneumoniae* strains.** Number of SNP was calculated by comparing the genome sequences to the reference (HS11286) genome. Background colors represent different number of pairwise SNPs, ranging from a minimum of 51 (white background) to a maximum of 24889 (ultramarine).
